# Genome-wide analysis of *OPR* family genes in *Vitis vinifera* and the role of *VvOPR1* in copper, zinc tolerance

**DOI:** 10.3389/fpls.2025.1509472

**Published:** 2025-02-26

**Authors:** Shuang-Hong You, Yuan-Ping Chen, Wen-Jing Shi, Xue Li, Zheng Wu, Quan-Hong Yao

**Affiliations:** ^1^ Fruit Research Institute, Chongqing Academy of Agricultural Sciences, Chongqing, China; ^2^ Biotechnology Research Institute, Shanghai Academy of Agricultural Sciences, Shanghai, China

**Keywords:** reductase, grapevine, gene family, copper and zinc stress, mechanism

## Abstract

12-oxo-phytodienoic acid reductase (OPR) is one of the key enzymes in the octadecanoid pathway, and it controls the last step of jasmonic acid (JA) biosynthesis. Although multiple isoforms and functions of *OPR*s have been identified in various plants, no *OPR* genes have been identified, and their possible roles in grapevine development and defense mechanisms remain unknown. In this study, nine *VvOPR* genes were identified from grapevine genome and classified into two subfamilies. Systematic analyses of the physical and chemical properties, the expression and structure of the *VvOPR* genes, promoter elements, and chromosome locations were performed via bioinformatics and molecular biology methods. In addition, we described the characterization of the *OPRI* gene *VvOPR1*, which was synthesized via a PCR-based two-step DNA synthesis quantification reverse-transcription (PTDS) method. *VvOPR1* expression is tissue-specific and induced by various stresses. The overexpression of *VvOPR1* in *Arabidopsis* and rice (OT) significantly increased tolerance to Cu, Zn stress, and Cu, Zn stress-induced restriction of the germination rate, root/shoot length and fresh weight was significantly alleviated in OT. In OT, *VvOPR1* enhanced the photosynthetic capacity, promoted ABA synthesis and the ABA-dependent stress response pathway, improved the antioxidation capacity by increasing the activities of ROS scavengers and the expression level of the related genes, while enhancing the accumulation of proline, AsA, GSH and reducing MDA and H_2_O_2_ levels. Moreover, *VvOPR1* reduced Cu^2+^, Zn^2+^ accumulation and translocation. Together, we first systematically characterized the grapevine OPR gene family and reported that *VvOPR1* responded to Cu, Zn stress in an ABA-dependent manner, and was quite independent of JA synthesis and signaling. All of the above results provide an important research basis and theoretical basis for further revealing the functions of *VvOPR* in grapevines in the future.

## 1 Introduction

The 12-oxo-phytodienoic acid reductase (OPR) is a key enzyme that catalyzes the conversion of 12-oxophytodienoic acid (OPDA) to 12-oxo-phytodienoic acid (OPC-8:0), a reaction that is a key process in jasmonic acid (JA) biosynthesis (Liechti and Farmer, 2006; Schaller, 2001). OPR enzymes are classified as flavin mononucleotide (FMN)-dependent oxidoreductases and belong to the old yellow enzyme (OYE) family, which is well represented in the yeast genome (Liu et al., 2020).

On the basis of substrate specificity, OPRs are split into two subgroups in dicots, subgroupI (OPRI) (e.g, AtOPR1 and AtOPR2) and subgroupII members (OPRII) (e.g, AtOPR3) which is involved in JA biosynthesis (Stintzi and Browse, 2000). Phylogenetic analysis of *OPR* genes in rice revealed that OPRs in monocots can be divided into five groups: sub. I-V (Li et al., 2011). *AtOPR*s can be activated by wounding, pathogens, cadmium, and hormone signaling molecules, such as JA, abscisic acid (ABA), salicylic acid (SA), and ethylene (ET) (Fattorini et al., 2018; Zheng et al., 2016). Recently, nine *IbOPR* genes were identified in sweet potato, and *IbOPR2* may play a crucial role in its response to salt stress by participating in JAs synthesis (Li et al., 2024). Seven *OPR* family genes (*CaOPR1-7*) were identified from the *Capsicum annuum* genome, the *CaOPR6* was highly similar to *AtOPR3*, and it played important roles in response to abiotic and biotic stresses (Nie et al., 2022). Five *OPR* family genes were identified in watermelon, expression analysis revealed that *ClOPR* genes, except for *ClOPR5*, were highly expressed in the flower and fruits, furthermore, the findings suggested *ClOPR2* and *ClOPR4* involved in red-light-induced defense against root-knot nematode (Guang et al., 2021).

The biochemical and physiological functions of *OPR*s have been reported in monocots such as rice (Wu et al., 2024), cotton (Liu et al., 2020; Hu et al., 2018), tomato (Breithaupt et al., 2006), maize (Zhang et al., 2005), pea (Matsui et al., 2004), wheat (Mou et al., 2019) and tea (Xin et al., 2017). The overexpression of *OsOPR1* improved the Cd tolerance of yeast cells by affecting the expression of antioxidant enzyme related genes and reducing Cd content in yeast cells (Wu et al., 2024). In upland cotton (*Gossypium hirsutum*), *GhOPR3* can be phosphorylated by *GhCPK33* at threonine-246 (Thr246) in peroxisomes, decreasing the protein level of GhOPR3, which consequently suppresses JA biosynthesis and reduces the resistance of cotton to *Verticillium dahliae* (Hu et al., 2018). In wheat (*Triticum aestivum*), the constitutive expression of *TaOPR2* can rescue the male sterility phenotype of the *AtOPR3* mutant, and these results suggest that *TaOPR2* is involved in the biosynthesis of JA (Wang et al., 2016a). In tomato (*Solanum lycopersicum*), the *SiOPR3* mutant plants exhibited increased susceptibility to *Botrytis cinerea* (Scalschi et al., 2015). In the tea (*Camellia sinensis*) plants, *CsOPR3* plays an important role in JA biosynthesis and defense against herbivorous insects (Xin et al., 2017). In maize (*Zea mays*), *ZmOPR7* and/or *ZmOPR8* are highly induced by wounding or treatment with JA, ethylene and ABA (Zhang et al., 2005), and the double mutant *ZmOPR7 ZmOPR8* exhibited delayed leaf senescence accompanied by reduced ethylene and ABA levels and a lack of anthocyanin pigmentation in brace roots (Yan et al., 2012). *OPRI* genes, which are not involved in the octadecanoid pathway (Schaller et al., 1998), are typically upregulated by pathogen invasion, wounding, and oxidative stress (Biesgen and Weiler, 1999; (Biesgen and Weiler, 1999; Strassner et al., 2002; Dong et al., 2013; Agrawal et al., 2003), events associated with ROS acceleration, so OPRIs are claimed to be concerned with antioxidant activity (Fitzpatrick et al., 2003; Trotter et al., 2006; Beynon et al., 2009). To date, the genome-wide identification of *OPR*s and the function of *OPRI* genes in grapevine have not been explicitly studied.

Grapevine (*Vitis vinifera*) is one of the most important economic crops in the world, and its growth and yield are restricted by various biotic and abiotic stresses in the filed. Cu, Zn are essential mineral elements for the normal growth and development of plants (Rehman et al., 2019; Adrees et al., 2015). However, long-term application of copper-based pesticides and fungicides (Manousaki et al., 2008; Nagajyoti et al., 2010), such as Bordeaux mixtures [with the Cu concentration at approximately 1.5 g/L] (Druart et al., 2012), which has been used intensively to control grapevine fungal diseases. Inorganic zinc (Zn) either alone or mixed with agrochemicals, such as fungicides against various diseases and organic fertilizers as sources or nutrients for grapevines is also often used in vineyards (Mirlean et al., 2007; Ramos and López-Acevedo, 2004). The accumulation of Cu, Zn in the vineyard soils has been increasing and often far beyond the required limits for normal grapevine growth (Yruela, 2005; Zalamena et al., 2015). Previous studies have explored the toxic effects of Cu, Zn on grapevines (Toselli et al., 2009; Juang et al., 2012; Miotto et al., 2014). However, the tolerance mechanism of the grapevines in response to Cu, Zn stress is not fully understood.

A genome-wide analysis of *OPR*s is essential for understanding the functions of the *OPR* gene family in grapevines and the possible roles of *VvOPRs* in development and defense against stress should be explored. In the present study, *OPR* family genes were firstly identified from the grapevine genome and nine *VvOPR*s which classified into two subfamilies were systematically analyzed. In addition, the *OPRI* gene *VvOPR1* was synthesize by PTDS (PCR-based two-step DNA synthesis quantification reverse-transcription) method, the function and mechanism of the grapevine abiotic stress-inducible *VvOPR1* gene was investigated via transgenic plants. Detailed analysis was carried out on the transgenic plants, and the results showed that the heterologous expression of *VvOPR1* in plants improved tolerance to Cu, Zn stress. All these results could contribute to screening more potential functional genes to improve tolerance against abiotic stresses in grapevines.

## 2 Materials and methods

### 2.1 Identification of homologous *OPR* genes in plants and sequence analysis of *VvOPR* family

Based on NCBI (http://blast.ncbi.nlm.nih.gov/Blast.cgi) database for the reported OPRs in *Arabidopsi*s (Biesgen and Weiler, 1999; Schaller and Weiler, 1997), the BLASTP and TBLASTP programs were used to search the Grape Genome Browser (http://www.genoscope.cns.fr/externe/GenomeBrowser/Vitis/) and Database (http://genomes.cribi.unipd.it/grape/), TAIR (http://www.arabidopsis.org/) and PlantTFDB (http://rice.plantbiology.msu.edu/) and a local protein database of homologous *OPR* genes in plants was constructed and *OPR* family genes in grapevine were obtained. A pairwise comparison of nucleotide and amino acid sequence similarity (%) between *VvOPR* family members was developed. The physical and chemical properties of the *VvOPR* family members, including the number of amino acids, molecular weight (MW), and isoelectric point (theoretical pI) were calculated using the online ExPaSy tool (http://www.ExPASy.org). The subcellular localization of the VvOPR proteins was predicted via the TargetP (https://services.healthtech.dtu.dk/services/TargetP-2.0/) and WoLF PSORT (https://wolfpsort.hgc.jp/) online servers.

### 2.2 Phylogenetic tree construction, gene structure, and promoter analysis

A paired comparison analysis between *VvOPR*s was developed via ClustalX software (2.1) (Larkin et al., 2007). *OPR*s from grapevine, rice and *Arabidopsis* were used to construct a phylogenetic tree via the neighbor-joining (NJ) method using MEGA software 6.0 (Tamura et al., 2013). The MEME program (http://meme-suite.org/tools/meme) was used to identify the conserved motifs of the VvOPR family members (Bailey et al., 2009). The gene structure display server program (GSDS 2.0) was used to illustrate the exon/intron organization of the *VvOPR* genes (Guo et al., 2007). The 2 kb upstream sequences of all of the *VvOPR* genes were considered as the promoters, and these sequences were extracted for the prediction of *cis*-regulatory elements via PlantCARE (Mou et al., 2019) Each position of the *VvOPR* gene on the grapevine chromosome was mapped via the software MapInspect.

### 2.3 Plant materials, growth conditions, and stress treatments

The plants (grapevine ecotype Maincure Finger, *Arabidopsis thaliana* ecotype Columbia L. and rice ecotype Wuyun Jing) were stored in our laboratory. The seeds of *Arabidopsis* and rice were surface-sterilized with 75% ethanol for 1.5 min, followed by 0.5% calcium hypochlorite for 20 min, and then rinsed at least three times with sterile distilled water. The sterilized seeds were placed on Murashige and Skoog (MS) medium with 1% agar and stratified in the dark at 4°C for 48 h. Then these plates were transferred to a controlled environmental chamber at a light intensity of ~120 μmol photons m^-2^s^-1^ at 22°C, maintained on a 16/8 h day/night cycle, with 70% relative humidity, and grown horizontally and/or vertically. After the rice seeds germinated, the seedlings were transferred into Hoagland’s liquid medium and/or pots filled with a 9:3:1 mixture of vermiculite/peat moss/perlite under the above conditions.

Cu/Zn treatments (Mikkelsen et al., 2012; Moravcová et al., 2018) were performed on grapevine, *VvOPR1*-overexpressing *Arabidopsis* and rice seedlings. Six-month-old grapevine seedlings were grown in Hoagland’s liquid medium supplemented with 10 μM CuSO_4_·5H_2_O or 0.1 mM ZnSO_4_·7H_2_O for 0, 3, 6, 9, 12 or 24 h. The whole grapevine plants from each treatment and/or the different organs (roots, stems and leaves) were sampled, frozen in liquid N_2_, and stored at -70 °C. The germination rate was determined when *VvOPR1*-overexpressing *Arabidopsis* seedlings were grown horizontally in MS medium supplemented with Cu (150 or 200 μM) or Zn (1.25 or 1.5 mM) for 2 weeks, respectively. The germination rate was calculated as the proportion of seedlings for which the radicle had begun to emerge from the seed coat. The morphological characteristics (root length and fresh weight) were determined at various Cu (50, 100 μM)/Zn (0.75, 0.87 mM) concentrations after *VvOPR1*-overexpressing *Arabidopsis* seedlings were oriented vertically in Petri dishes containing MS medium incubated for 14 days. A Cu/Zn solution of deionized water was added to Hoagland’s liquid medium to obtain concentrations of Cu 0, 200, 600 and 900 μM/Zn 0, 2.5, 5.0 and 7.5 mM, and two-week-old transgenic rice seedlings were used to detect Cu/Zn tolerance. Then, 4-week-old transgenic rice seedlings were subjected to Cu (750 μM), Zn (7.5 mM), Cu+Zn (750 μM+7.5 mM) treatment for 5 d, and immediately transferred to liquid nitrogen and then a freezer (-80 °C) until further physical and chemical analyses.

### 2.4 Analysis of gene expression

Three seedlings were selected randomly from each treatment, and frozen grapevine and rice tissues (0.2 g) were ground thoroughly in liquid nitrogen using a mortar and pestle, respectively. Grapevine RNA extraction was performed via the modified CTAB method (Wang et al., 2011), and total RNA from *VvOPR1*-overexpressing rice was extracted from the disrupted tissues using the extraction medium (Qiagen) according to the manufacturer’s instructions. The RNA concentration was measured using a NanoDrop 2000 (Thermo Fisher Scientific, Waltham, MA, USA) and RNA integrity was assessed using an RNA Nano 6000 Assay Kit in conjunction with an Agilent Bioanalyzer 2100 system (Agilent Technologies, Santa Clara, CA, USA). The genomic DNA was removed by incubating the RNA sample in gDNA wipeout buffer (Qiagen) at 42 °C for 2 min before complementary DNA (cDNA) synthesis. First-strand cDNA was synthesized from 5 μg of total RNA with the QuantiTect Reverse Transcription Kit (Qiagen) in a 20 μL reaction volume according to the manufacturer’s instructions and it was stored at -20 °C until it was used in real-time quantitative PCR assays.

Quantitative real-time PCR was performed with a 7500 Fast Real-Time PCR system (Applied Biosystems), using SYBR Green chemistry. PCRs were conducted in a final volume of 20 μL containing 2 μL of a 1:10 dilution of cDNA sample, 10 μL of iQ SYBR Green Supermix, and 0.5 μmol/L of each primer. The thermal cycling conditions included initial denaturation at 95 °C for 30 s, followed by 40 cycles of 95 °C for 5 s, and 65 °C for 15 s for annealing and extension. PCR efficiency was checked for all primers used for gene expression analyses (**Supplementary Table S1**). Only primers with higher amplification efficiency (>90%) were used in this experiment. The *VvActin* and *OsUBQ5* genes were used as references in grapevine and rice seedlings, respectively. The relative expression levels of the target genes (antioxidative enzyme-encoding genes and ABA- and JA-related genes) were determined via the standard 2^−ΔΔCT^ method of Livak and Schmittgen (2001). All samples were tested in duplicate for the reference genes as well as for the genes of interest.

### 2.5 Synthesis of *VvOPR1*, vector construction and *Arabidopsis*/rice transformation

The full-length of *VvOPR1* (XP_002281119) gene was artificially biosynthesized via PTDS method (Xiong et al., 2004). The synthesized *VvOPR1* was cloned into the TA cloning vector Simple *pMD-18* (*pMD18-T*) and the high integrity of the construct was confirmed by sequencing. The coding sequence was subsequently excised from the *pMD18-T* vector and inserted into the *pCAMBIA1301* vector under the control of the CaMV 35S promoter. The constructed plant expression vectors were transformed into *Agrobacterium* (strain EHA105) via the freeze-thaw procedure, and strains containing this vector were transformed into the rice cultivar Wuyun Jing according to the methods of Tang (Tang et al., 2017). *VvOPR1*-overexpressing *Arabidopsis* was constructed via the floral dip method (Zhang et al., 2006). Positive transformants were selected via hygromycin (50 μg/ml) resistance and confirmed by PCR using the specific primers. In addition, *VvOPR1* was transformed into the *Arabidopsis NCED1* mutant which is defective in ABA synthesis to construct *VvOPR1/NCED1* lines.

### 2.6 Stress tolerance analysis

Three *VvOPR1*-overexpressing *A. thaliana* seedlings were selected randomly from each treatment, and their morphological characteristics (germination rate, root length and fresh weight) were measured and compared. Four-week-old OT and WT rice seedlings grown in Hoagland’s liquid medium supplemented with Cu (750 μM), Zn (7.5 mM), Cu+Zn (750 μM+7.5 mM) for five days were sampled for physical and chemical analyses. The Chl and Car contents were estimated via the Lichtenthaler (1987) and Wellburn (1994) methods. The root activity and MDA was determined as described by Wang et al. (2016b). The content of H_2_O_2_ was determined via the method of Zhou et al. (2018) with slight modifications. The free proline content was determined according to the methods of Bates et al. (1973) and Song et al. (2011). The antioxidants and antioxidant enzymes in the AsA-GSH cycle were detected refer to previous studies (Zhou et al., 2018; Geng et al., 2018; Thounaojam et al., 2012).

### 2.7 JA and ABA analysis

Rice leaves were extracted for ABA (Lou and Baldwin, 2003) and JA (Hung and Kao, 2003) analysis following previous studies with slight modifications. The crude extract was centrifuged and passed through polyvinylpyrrolidone column and C18 cartridges to remove plant pigments and other nonpolar compounds. JA extraction was analyzed by GC-MS with a doubly labeled internal standard ([1,2-^13^C]-JA). The ABA eluates were then concentrated to dryness by vacuum evaporation and resuspended in Tris-buffered saline. Afterwards, ABA was subsequently determined spectrophotometrically at 405 nm with an ABA immunoassay detection kit (model PGR-1; Sigma-Aldrich, St. Louis MO, USA).

### 2.8 Evaluation of the Cu^2+^/Zn^2+^ concentration in different parts of plants grown in Hoagland’s liquid medium

For Cu^2+^/Zn^2+^ determination, both the aerial and root parts of the Cu/Zn-treated OT and WT seedlings were harvested, carefully rinsed with distilled water, oven dried at 80 °C for two days and ground (Li et al., 2017). The dried plant parts were digested with HNO_3_ (11N) at 200°C for 10 h, and the digested samples were then diluted with HNO_3_ (0.1 N) until a clear liquid formed. The filtrate was analyzed via ICP-OES (GBC INTEGRAXL, Australia) and standard solutions from MERCK and Analytika Praha were used to determine the metal content. The Cu^2+^ content was determined at a wavelength of 324.754 nm, and the Zn^2+^ content was measured at 213.856 nm (Tanhan et al., 2007). The TF, TI and AR for Cu^2+^/Zn^2+^ were detected using the following formulas developed by previous studies (Shu et al., 2002; Khan et al., 2015) based on the dry weight of the seedlings (**Supplementary Tables S1, S2**).

### 2.9 Statistical analysis

The treatments of the experiments were arranged in a completely randomized block design, and three replicates were performed for each treatment. The data analysis was performed statistically using analysis of variance (ANOVA) via SPSS 17.0 and the data are presented as the means ± SEs (n=3). A statistically significant difference (p<0.05) was calculated by the least significant difference (LSD) test and is shown in the figures.

## 3 Results

### 3.1 Identification of homologous *OPR* genes in plants and the chromosomal position of *VvOPR* family genes

**Figure 1:**
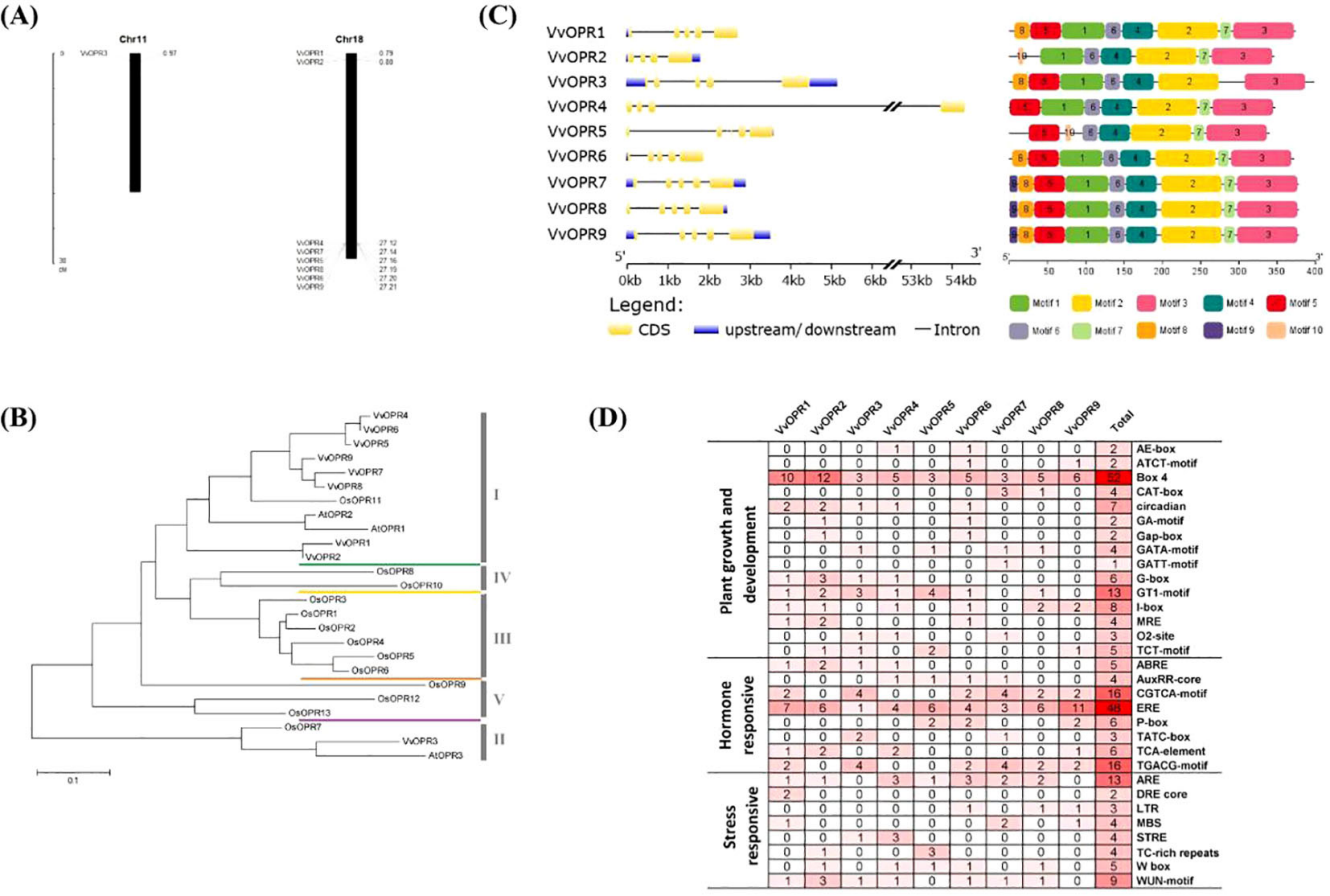
Genome-wide Analysis of *VvOPR* Family Genes. **(A)** The chromosomal positions of the *OPR* genes in grapevine. **(B)** The phylogenetic tree of OPR proteins in grapevine, *Arabidopsis* and rice. **(C)** Gene structure and conserved motifs of VvOPR family members. **(D)** Frequency and function of *cis*-regulatory elements (*CRE*s) in the promoter regions of *VvOPR* genes. 1: Plant growth and development; 2: Hormone responsive; 3: Stress responsive.

Based on NCBI database and literature, 121 homologous *OPR* genes were identified from 12 plant species representing six major plant lineages, including algae, moss, pteridophyta, gymnosperm, monocot and dicot (**Table 1**). Nine *VvOPR*s (*OPRs* from *Vitis vinifera*) were identified from grapevine and named *VvOPR1*~*9*. The putative *VvOPR* genes were predicted to encode proteins ranging from 340 to 398 amino acids in length, the molecular weight (Mw) and isoelectric point (pI) of the VvOPR proteins ranged from 37.63 to 43.71 KDa and from 5.45 to 8.23, respectively (**Table 2**). In terms of amino acid composition, positive amino acids accounted for the greatest percentage, followed by aliphatic amino acids and aromatic acids, and negative amino acids accounted for the lowest percentage (**Table 2**). Almost all of the VvOPR proteins were predicted to accumulate in the cytoplasm (Cyto). The chromosomal positions of the *VvOPR* genes revealed that OPR genes were distributed in clusters, the *VvOPR* genes were distributed across two chromosomes with different densities (**Figure 1A**), the putative *VvOPR3* genes were mapped to chromosome 11, and the other eight *VvOPR*s were mapped to chromosome 18. *VvOPR1~2* mapped to the top of chromosome 18, and *VvOPR4~9* mapped to the bottom of chromosome 18.

**Table 1:** *OPR* or *OPR*-like genes in representative plants.

Lineage	Plant	Number	Nomenclature
Algae	*Chlamydomonas reinhardtii*	3	*CrOPR*
Moss	*Physcomitrella patens*	6	*PpOPR*
Pteridophyta	*Selaginella moellendorffii*	6	*SmOPR*
Gymnosperm	*Picea sitchensis*	3	*PsOPR*
Monocots	*Oryza sativa*	13	*OsOPR*
	*Zea mays*	8	*ZmOPR*
	S*orghum bicolor*	13	*SbOPR*
	*Triticum aestivum*	48	*TaOPR*
Dicots	*Arabidopsis thaliana*	3	*AtOPR*
	*Lycopersicum esculentum*	3	*LeOPR*
	*Vitis vinifera*	9	*VvOPR*
	*Pisum linnaeus*	6	*PlOPR*

**Table 2:** Characteristic features of *VvOPR* family members.

Gene	Locus Id	Size (aa)	Mw (KDa)	pI	Positive amino acid/%	Negative amino acid/%	Aliphatic amino acid/%	Aromatics amino acid/%	Subcellular localization	Protein GRAVY
*VvOPR1*	VIT_218s0122g01160	374	41.73	5.70	17	10	14	13	Cyto/-	-0.507
*VvOPR2*	VIT_218s0122g01170	346	38.58	5.45	17	11	13	13	Mito/Mito	-0.449
*VvOPR3*	VIT_211s0016g01230	398	43.71	8.23	20	9	12	10	Chlo/Chlo	-0.271
*VvOPR4*	VIT_218s0041g02010	347	38.56	5.72	19	10	13	12	Mito/Mito	-0.367
*VvOPR5*	VIT_218s0041g02040	340	37.63	5.41	21	9	12	12	Cyto/-	-0.276
*VvOPR6*	VIT_218s0041g02070	372	41.27	6.28	20	10	13	12	Cyto/Mito	-0.326
*VvOPR7*	VIT_218s0041g02020	379	42.16	5.84	21	9	13	12	Cyto/-	-0.407
*VvOPR8*	VIT_218s0041g02060	379	41.90	6.02	21	9	13	12	Cyto/-	-0.362
*VvOPR9*	VIT_218s0041g02080	379	41.93	5.88	20	10	12	12	Cyto/-	-0.324

Cyto, cytoplasm; Mito, mitochondrion; Chlo, chloroplast; -, any other location.

### 3.2 Phylogenetic tree construction and sequence analysis of *VvOPR* genes

To evaluate the evolutionary relationships within the *OPR* gene family in grapevine, a rooted maximum-likelihood (ML) phylogenetic tree with 25 *OPR* genes from monocots (rice) and dicots (*Arabidopsis* and grapevine) (**Figure 1B**) was established. Phylogenetic analysis revealed that the *OPR* gene family can be subdivided into five well-conserved subfamilies, and these subfamilies are numbered sub.I to V. All *OPR* genes from grapevine, *Arabidopsis* and several *OPR*s from rice were grouped into sub.I and II, indicating that all *OPR* genes from angiosperms shared a common ancestor before the divergence between dicots and monocots. Several *OPR*s from rice were grouped into sub.III to V, showing that lineage-specific expansion and divergence events occurred in monocots after divergence from dicots and sub.III to V were generated exclusively in monocots. To explore different selective constraints on duplicated *VvOPR* genes, the *Ka* (synonymous nucleotide substitution), *Ks* (non-synonymous nucleotide substitution) and *Ka/Ks* ratios for each pair of duplicated *VvOPR* genes were calculated, and seven duplicated pairs of paralogous genes were identified in the nine *VvOPR*s. The results showed that the ratios of *Ka/Ks* for two duplicated pairs were <1, ranging from 0.1986 to 0.6999 (**Table 3**). These results revealed that the gene duplication events occurred in these genes and that the functions of the duplicated genes did not diverge during genome evolution after the duplication events.

**Table 3:** *Ka* and *Ks* values of paralogous gene pairs of *VvOPR*.

Seq_1	Seq_2	*Ka*	*Ks*	*Ka*/*Ks*
*VvOPR1*	*VvOPR2*	0.0231	0.0333	0.6920
*VvOPR4*	*VvOPR5*	0.0301	0.0539	0.5586
*VvOPR4*	*VvOPR6*	0.0115	0.0202	0.5694
*VvOPR5*	*VvOPR6*	0.0574	0.0820	0.6999
*VvOPR7*	*VvOPR8*	0.0277	0.0552	0.5015
*VvOPR7*	*VvOPR9*	0.0355	0.1452	0.2448
*VvOPR8*	*VvOPR9*	0.0275	0.1387	0.1986

To further detect the origin and evolution of the *VvOPR* family genes, the nucleotide and amino acid sequences of the nine *VvOPR*s were blasted via pairwise alignment, respectively (**Table 4**). The results revealed that the level of nucleotide and amino acid sequence identity among the *VvOPR*s varied from 28.3% to 98.1% and 44.4% to 97.4%, respectively. Five pairs of *VvOPR*s showed extremely high homology (the level of sequence identity between *VvOPR*s was above 90%), as follows: *VvOPR4/VvOPR6* > *VvOPR1/VvOPR2* > *VvOPR7/VvOPR8* > *VvOPR8/VvOPR9* > *VvOPR7/VvOPR9*. The level of nucleotide/amino acid identity between *VvOPR3* and the other eight *VvOPR*s varied from 28.3% to 36.7%/44.4% to 51.9%. These results indicated that *VvOPR3* may have undergone rapid evolution and functioned differently from the other eight *VvOPR* genes.

**Table 4:** Pairwise comparison of nucleotide and amino acid sequence identity (%) between *OPR* family members in grapevine.

	*VvOPR1*	*VvOPR2*	*VvOPR3*	*VvOPR4*	*VvOPR5*	*VvOPR6*	*VvOPR7*	*VvOPR8*	*VvOPR9*
*VvOPR1*	–	97.2	36.4	44.3	40.2	43.8	45.5	47.0	47.2
*VvOPR2*	95.4	–	36.7	45.8	41.3	45.6	46.6	48.2	48.2
*VvOPR3*	48.4	50.3	–	33.2	28.3	33.8	32.3	32.5	33.9
*VvOPR4*	62.5	64.5	50.7	–	87.3	98.1	75.4	77.0	78.1
*VvOPR5*	55.9	57.6	44.4	85.0	–	89.4	68.6	70.4	70.3
*VvOPR6*	62.4	64.7	51.9	97.4	87.9	–	73.8	75.4	76.1
*VvOPR7*	62.6	66.2	50.1	81.3	74.7	80.6	–	95.8	92.8
*VvOPR8*	64.4	68.2	51.2	83.0	76.5	82.8	93.9	–	94.5
*VvOPR9*	64.2	67.9	51.5	84.4	78.2	84.7	92.1	94.5	–

The data above ‘-’ represented the amino acid sequence similarity, and data below ‘-’ represented the nucleotide similarity. Coding region nucleotide (upper portion of matrix) and amino acid (bottom portion of matrix) sequence pairwise comparisons (% similarity) between grapevine OPR genes.

### 3.3 Analysis of the *VvOPR* gene structure and *cis*-regulatory elements of its promoter

An exon-intron analysis was performed by comparing the predicted coding sequence (CDS) with the sequence of the *VvOPR* genes. As shown in **Figure 1C**, the VvOPR family members presented a highly conserved exon-intron structure, which contained 4~6 exons and 3~5 introns. *VvOPR4* shared the longest intron sequence, an untranslated region of 53 kb. In addition, the nine VvOPR proteins shared at least four conserved motifs (motifs 6, 4, 2 and 3), which showed the same alignment. VvOPR7, VvOPR8 and VvOPR9 were completely consistent and motif 9 is characteristic of the three VvOPR proteins. Motif 7 was only deleted in VvOPR3. Phylogenetic analysis revealed that VvOPR members in the same branch were structurally conserved, indicating that motif 7 may play an important role in the evolution and function of *VvOPR*s. Taken together, the fact that the VvOPR proteins not only share very similar intron/exon structures but also contain common motifs supports their close evolutionary relationships and membership in the same subfamily. All of these results suggested that the strategies of *VvOPR* genes classification were relevant and reliable.

To further understand the biological regulation of the *VvOPR* genes, the *cis*-regulatory elements (*CRE*s) in the upstream of these genes were identified via the PlantCare database. The *cis*-elements included plant growth and development elements, hormone responsive elements, and environmental stress responsive elements and so on (**Figure 1D**). The frequency of these elements in the regulatory region of each corresponding gene, as well as their overall frequency in family members, is very diverse. Most *CRE*s involved in plant growth and development, such as Box 4 (52 times), GT1-motif (13 times) and I-box (8 times) are related to light-responsive elements. Hormone-responsive elements include those involved in ethylene response (ERE) and methyl jasmonate (MeJA). Classic plant hormones such as SA, ETH and MeJA are involved in the regulation and integration of plant immune responses against pests and pathogens.

### 3.4 *VvOPR1* expression is tissue-specific and induced by various stresses

**Figure 2:**
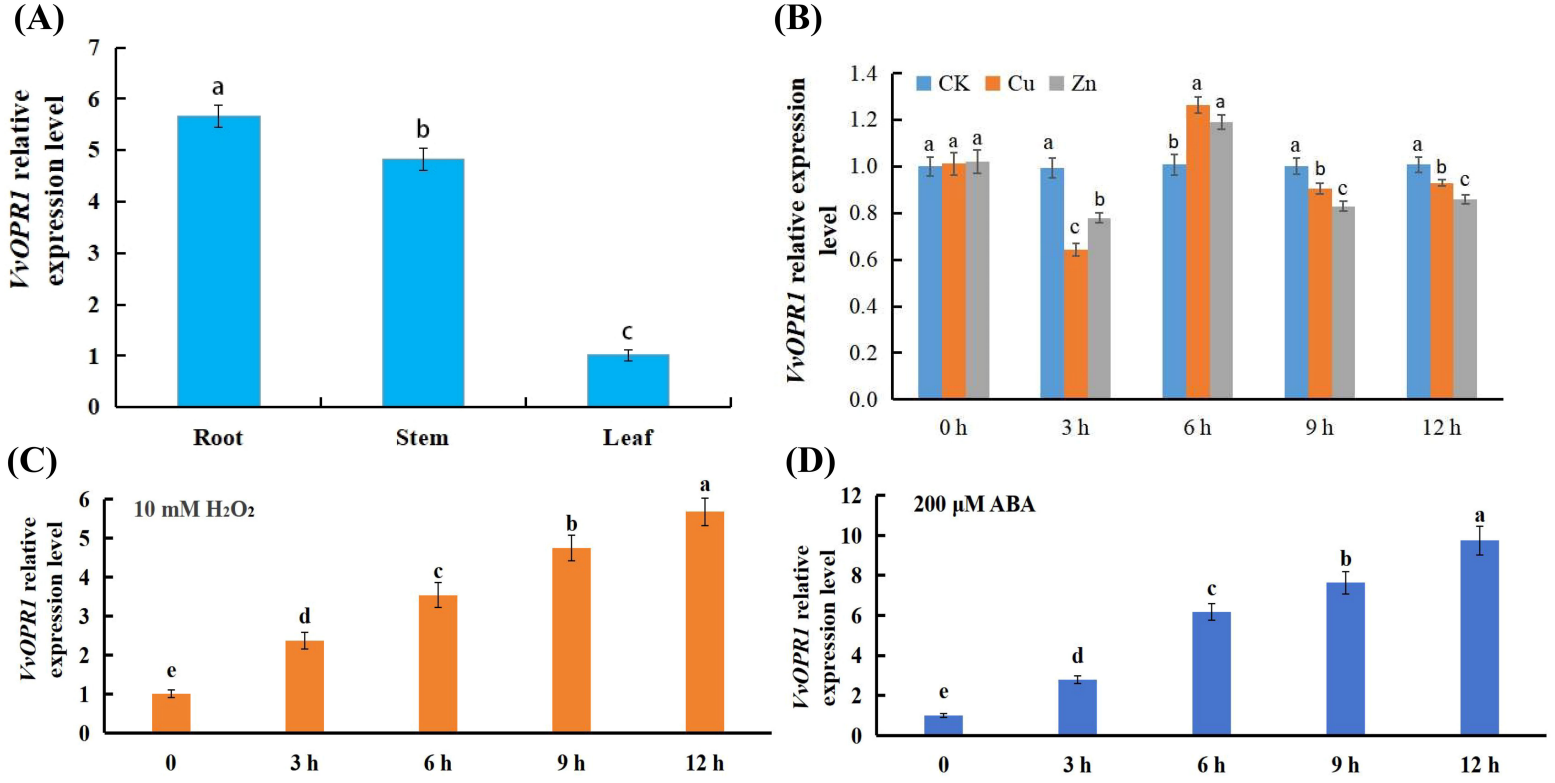
Expression analysis of the *VvOPR1* gene and *VvOPR1* is induced by various abiotic stresses in grapevine leaves. **(A)** The qRT-PCR analysis of the expression level of *VvOPR1* in different tissues of grapevine during seedling stage. **(B-D)** The qRT-PCR analysis of *VvOPR1* expression in grapevine leaves subjected to **(B)** Cu, Zn; **(C)** 10 mM H_2_O_2_; **(D)** 200 μM ABA stress, respectively. Different lowercase letters indicate significant differences (P<0.05) in the expression level between the different tissues of grapevine **(A)** and between different time points subjected to various stress treatments, respectively **(B-D)**.

Different tissues (roots, stems and leaves) of six-month-old grapevine (Maincure Finger) seedlings were sampled, and real-time quantitative PCR (qRT-PCR) was used to examine the *VvOPR1* expression profile. The results revealed that various expression levels of *VvOPR1* could be detected in all of the tissues tested, and the expression level of *VvOPR1* was the highest in the roots and the lowest in the leaves (**Figure 2A**). As shown in **Figure 2B**, under Cu, Zn stress, *VvOPR1* expression rapidly decreased throughout the 3 h period, and the expression of *VvOPR1* was higher under Zn stress than under Cu stress. Then, *VvOPR1* expression increased and peaked at 6 h, and the expression of *VvOPR1* level was higher under Cu stress than under Zn stress from 6 h to 12 h, the plateau lasted from 9 h to 12 h testing period. Exposure to excess Cu, Zn stress could stimulate the overproduction of reactive oxygen species (ROS) and abscisic acid (ABA), the former impairs antioxidant defense systems and the latter triggers downstream stress-responsive pathways (Mittler et al., 2011). Here, the expression level of *VvOPR1* almost mirrored the same trend after exposure to 10 mM H_2_O_2_ and 200 μM ABA, the expression level of *VvOPR1* increased gradually from 0 h to 12 h under the treatment conditions (**Figures 2C, D**). All these results suggest that the expression pattern of *VvOPR1* is tissue-specific and that its expression can be induced by various stresses.

### 3.5 The transformation of *VvOPR1* and the confirmation of putative transgenic seedlings

**Figure 3:**
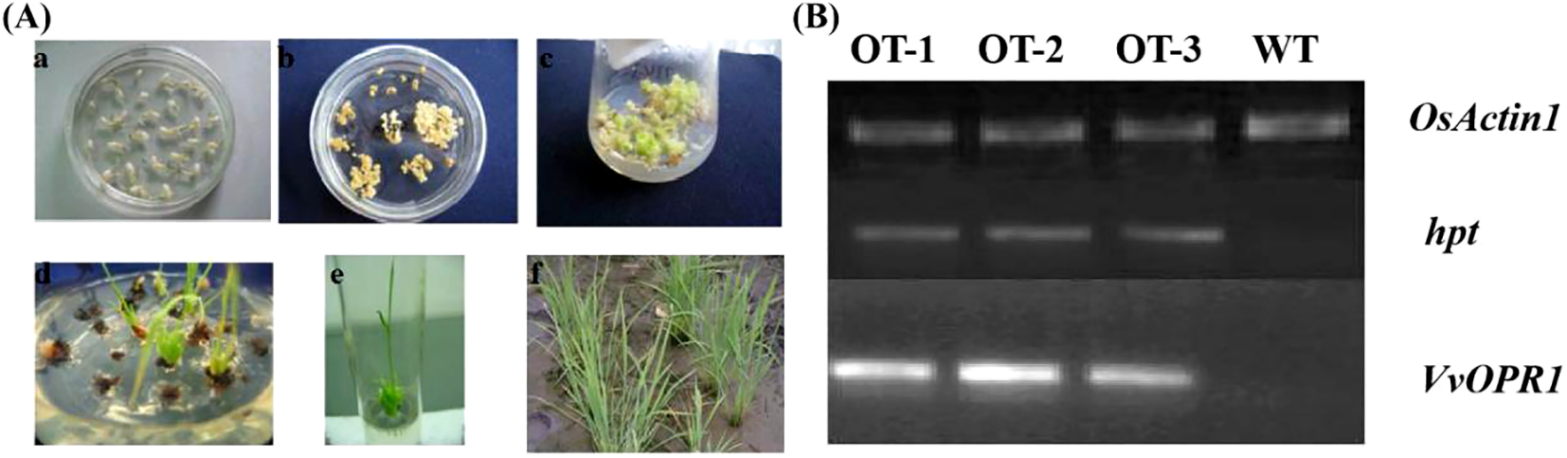
The transformation and *VvOPR1* PCR in rice seedlings. **(A)** The procedure of *Agrobacterium*-mediated genetic transformation of rice calli, including: (a) Calli induction; (b) First screening; (c) The second screening; (d) Differentiation; (e) Taking root; (f) Transplantation. **(B)** RT-PCR analysis of the *VvOPR1* gene fragments from the transgenic (OT-1, OT-2, and OT-3) and wild-type (WT) plants, *OsActin1* gene was used as a reference.

*VvOPR1* was synthesized via the PTDS method and transferred into both *Arabidopsis* and rice (**Figure 3A**). Several independent lines of transgenic seedlings (T1 generation) were subsequently generated. After selecting for hygromycin resistance, PCR was performed (T3 generation) (**Figure 3B**), the putative transgenic plants were confirmed, and several transgenic T3 plant lines (OT-1, OT-2 and OT-3) were chosen for further experiments. The phenotypes of the transgenic plants (T3 generation) were similar to those of the wild-type (WT) plants both on MS plates and in soil pots. These findings indicated that overexpression of *VvOPR1* in *Arabidopsis* and rice caused no visible morphological changes.

### 3.6 Effects of Cu, Zn treatments on visual appearances and growth of OT seedlings

**Figure 4:**
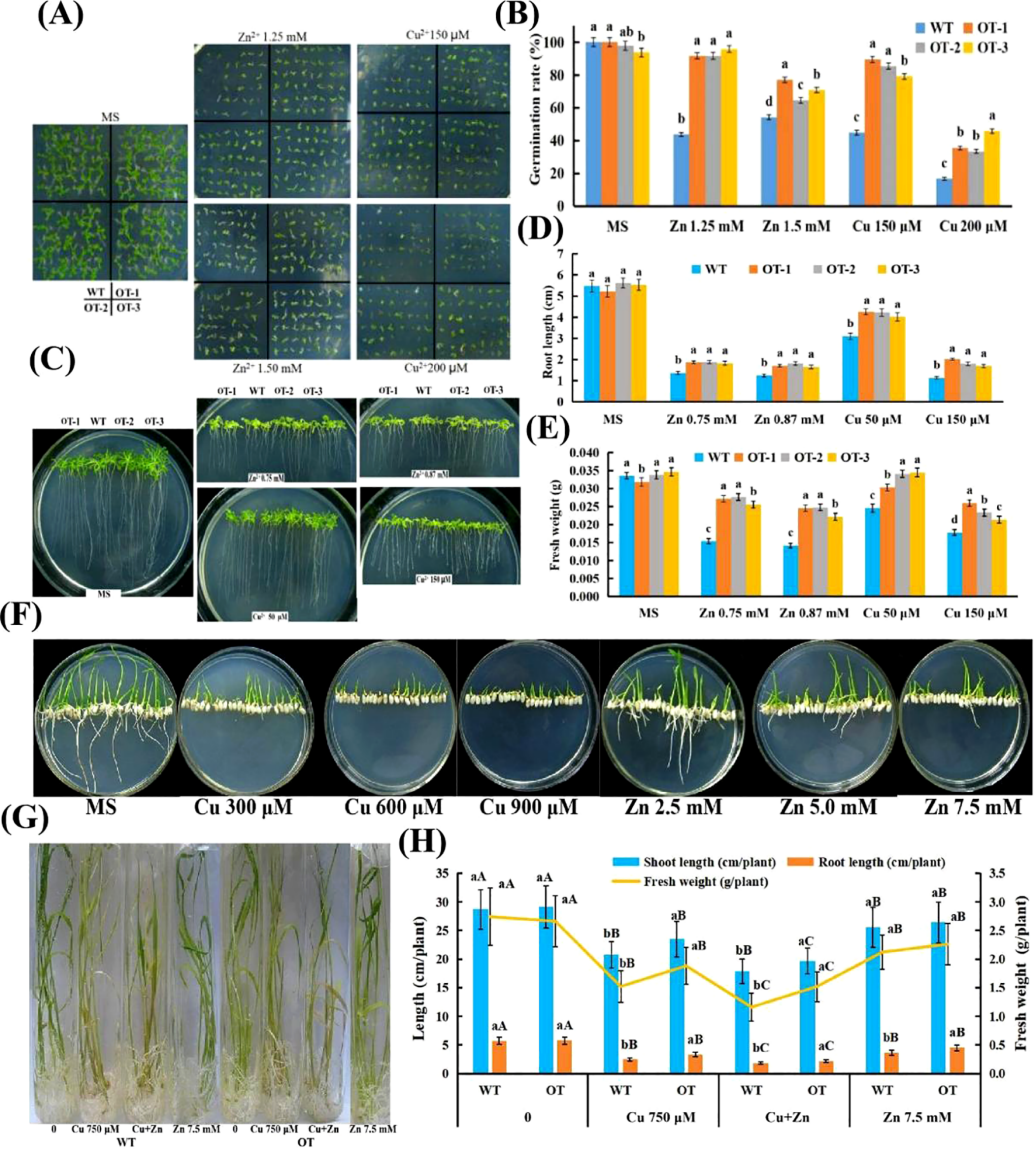
Performance of WT and OT *Arabidopsis/*rice under normal and Cu, Zn stress conditions. **(A)** Left panel, seedlings reared under normal conditions for three weeks; right panel, seedlings exposed to 125, 150 mM Zn and 150, 200 μM Cu for three weeks, respectively. **(B)** The germination rates of plants after grown horizontally on the MS medium with different Cu, Zn concentrations for four weeks. **(C)** *VvOPR1* over-expressing and WT plants grown vertically in MS medium with or without Cu, Zn for three weeks, respectively. **(D)** Comparison of root length between OT *Arabidopsis* linesand WT seedlings presented in panel **(C)**. **(E)** Comparison of fresh weight between OT lines and WT seedlings presented in panel **(C)**. **(F)** Visual aspects of Cu/Zn-toxicity on rice seedlings. WT and OT rice seedlings grown vertically for two weeks on MS medium with different Cu, Zn concentrations. **(G)** Visual aspects of Cu, Cu+Zn and Zn-toxicity on rice seedlings. The four-week-old WT and OT rice seedlings grown in Hoagland’s liquid medium with 750 μM Cu, 750 μM Cu+7.5 mM Zn and 7.5 mM Zn for five days. **(H)** Effects of Cu, Cu+Zn and Zn on shoot length, root length and fresh weight of WT and OT rice seedlings. Each data point is the mean value ± SD of three replicates. The statistical significance was determined by Duncan’s multiple comparison tests. Different lowercase letters indicate significant differences (P<0.05) between WT and OT rice seedlings in the same treatment, and different capital letters indicate significant difference (P<0.05)among different treatments in WT and OT, respectively.

The performance of WT and OT *Arabidopsis* seedlings under both normal and Cu and Zn stress conditions was compared to investigate whether the overexpression of *VvOPR1* could potentiate the tolerance of OTs to Cu, Zn stress (**Figures 4A-E**). No significant difference in growth performance was detected between the two types of seedlings in the absence of stress, but all seedlings from each type were obviously suppressed when they were grown for three weeks in the medium containing Cu, Zn, and the suppression was more severe in the WT seedlings than in the OT seedlings (**Figures 4A-E**). The germination rate of OT *Arabidopsis* seeds was nearly twofold higher compared with that of WT seeds on medium supplemented with 150, 200 μM Cu. A similar pattern was observed for OT seeds under 1.2 mM Zn stress condition (**Figures 4A, B**). Exposure to Zn stress, compared with that of WT seedlings, the root length of OT seedlings increased by 32% to 46%, whereas the fresh weight increased by 56% to 80% (**Figures 4C, D, E**). Under Cu stress, the root length increased by 30% in the 50 μM Cu treatment, however, the root length increased from 50% to 78% in the OT lines compared with that in the WT plants under the 150 μM Cu treatment (**Figures 4C, D**), whereas the fresh weight increased by from 20% to 45% (**Figures 4C, E**).

The effects of Cu, Zn stress on OT rice seedlings grown in MS medium and Hoagland’s liquid medium were detected (**Figures 4F–H**). When treated with Cu, WT rice showed neither any roots nor any cotyledons, while several cotyledons were detected in OT grown in MS medium (**Figure 4F**). When Zn stress was imposed on the rice seedlings, all of the WT presented no any roots and much fewer cotyledons than the OT did, whereas all of the Zn-treated OT seedlings showed several leaves and roots that became shorter with increasing Zn concentration in the MS medium (**Figure 4F**). After exposure to Hoagland’s liquid medium containing Cu, Zn for 5 days, several morphological disturbances were detected in the OT and WT. Visible toxicity symptoms such as chlorosis, necrosis and rolling of the leaves were more obvious upon Cu and Cu+Zn impositions (**Figure 4G**), and the roots of the Cu- and Cu+Zn-stressed seedlings became reddish, and their health deteriorated compared with that of the control seedlings (**Figure 4G**). Both the OT and WT seedlings exhibited a decrease in shoot length, root length and fresh weight significantly when they were exposed to 750 μM Cu solution (**Figure 4G**); to some degree, the greatest reduction in shoot length, root length and fresh weight occurred in the seedlings subjected to Cu+Zn stress (**Figures 4G, H**). Compared with those grown under Cu, Cu+Zn conditions, the Zn-stressed seedlings presented fewer toxic effects and retained a similar leaf color (**Figures 4G, H**). Together, compared with the WT seedlings, the OT presented significantly higher shoot length, root length and fresh weight under stress conditions, indicating that the tolerance of the OT seedlings to Cu, Zn stress was greater than that of the WT seedlings.

### 3.7 Assays of the photosynthetic activities of *VvOPR1*-overexpressing rice subjected to Cu, Zn stress

Chlorophyll fluorescence has been routinely used to monitor the photosynthetic performance of plants noninvasively and to screen plants for tolerance to environmental stresses (Baker and Rosenqvist, 2004). To determine whether the chlorophyll content of OT is affected by Cu, Zn stress, the chlorophyll (Chla) and carotenoid (Car) contents were detected. As shown in **Table 5**, there was no significant difference in the Chla, Chlb, Chla/Chlb or Car contents between WT and OT under normal conditions. In contrast, following the Cu, Zn and Cu+Zn treatments, the Chla, Chlb, Chla/Chlb and Car contents decreased in both the WT and OT seedlings compared with those under the control conditions, but the Chla, Chlb and Chla/Chlb values in the leaves of the OT were significantly higher than those in the WT, whereas the Car content in the OT was lower than that in the WT. The Chla, Chlb and Car contents in the leaves of the rice seedlings treated with 7.5 mM Zn were greater than those in the rice lines treated with 0.75 mM Cu and 0.75 mM Cu+7.5 mM Zn. Cu, Zn damage to the OT plants was evaluated both as photosystem II (PSII) stability and as injury to the whole plant. Fv/Fm (variable fluorescence/maximal fluorescence) was used to estimate the quantum yield of PSII photochemistry. The F_0_ (minimal fluorescence), Fm and Fv of WT and OT plants were measured, and no significant difference was detected between WT and OT plants under control condition. The values of OT were higher than those of WT seedlings after treatment with 0.75 mM Cu, 7.5 mM Zn and 0.75 mM Cu+7.5 mM Zn, respectively (**Table 6**). In particular, the combined Cu, Zn treatment markedly inhibited PSII, as indicated by a decrease in the Fv/Fm value. Together, these results are consistent with the hypothesis that plants overexpressing *VvOPR1* are more tolerant of photoinhibition than are WT seedlings.

**Table 5:** Effect of different metals on the chlrophyll content in rice leaves.

Treatment	Chla (mg/g FW)		Chlb (mg/g FW)		Car (mg/g FW)		Chla/Chlb	
	WT	OT	WT	OT	WT	OT	WT	OT
0.00	0.74 ± 0.03 aA	0.81 ± 0.09 aA	0.38 ± 0.04 aA	0.39 ± 0.05 aA	0.21 ± 0.02 bA	0.19 ± 0.04 bA	1.95 ± 0.12 aA	2.08 ± 0.19 aA
Cu 0.75mM	0.43 ± 0.11 cB	0.57 ± 0.02 cA	0.27 ± 0.01cB	0.32 ± 0.06 bA	0.13 ± 0.02 dA	0.12 ± 0.00 dA	1.59 ± 0.11 cB	1.78 ± 0.16 cA
Zn 7.5 mM	0.55 ± 0.04 bB	0.67 ± 0.03 bA	0.31 ± 0.02 bB	0.36 ± 0.09 aA	0.17 ± 0.01cA	0.14 ± 0.01 cB	1.83 ± 0.15 bA	1.86 ± 0.12 bA
Cu+Zn	0.31 ± 0.02 dB	0.45 ± 0.02 bA	0.21 ± 0.01 dB	0.28 ± 0.03 cA	0.26 ± 0.03 aA	0.23 ± 0.05 aB	1.47 ± 0.13 dB	1.61 ± 0.14 dA

Each data point is the mean value ± SD of three replicates, the statistical significance was determined by Duncan’s multiple comparison tests. Different capital letters indicate a significant difference (p<0.05) between the WT and OT in the same row. Different lower cases indicate a significant difference (p<0.05) of different treatments in the same column.

**Table 6:** Effect of different metals on fluorescence dynamics of rice.

Treatment	Fo		Fm		Fv		Fv/Fm	
	WT	OT	WT	OT	WT	OT	WT	OT
0	308 ± 19.43 aA	316 ± 24.56 aA	1495 ± 117.64 aA	1523 ± 143.76 aA	1198 ± 112.45 aA	1246 ± 145.76 aA	0.80 ± 0.07 aA	0.82 ± 0.07 aA
Cu 0.75mM	283 ± 23.16 aB	298 ± 20.85 aA	787 ± 54.56 cB	876 ± 67.45 cA	485 ± 32.56 cB	594 ± 46.76 cA	0.62 ± 0.06 cB	0.68 ± 0.05cA
Zn 7.5mM	292 ± 24.27 aB	309 ± 21.35 aA	968 ± 78.52 bA	995 ± 78.42 bA	687 ± 43.56 bB	758 ± 89.65 bA	0.71 ± 0.06 bB	0.76 ± 0.06 bA
Cu+Zn	235 ± 22.78 bB	289 ± 18.67 aA	695 ± 53.23 dB	776 ± 103.56 dA	381 ± 21.56 dB	473 ± 37.87 dA	0.55 ± 0.03 dB	0.61 ± 0.05cA

Each data point is the mean value ± SD of three replicates, the statistical significance was determined by Duncan’s multiple comparison tests. Different capital letters indicate a significant difference (p<0.05) between the WT and OT in the same row. Different lower cases indicate a significant difference (p<0.05) of the column.

### 3.8 Cu, Zn-induced ROS accumulation in response to oxidative damage in OT rice leaves

**Figure 5:**
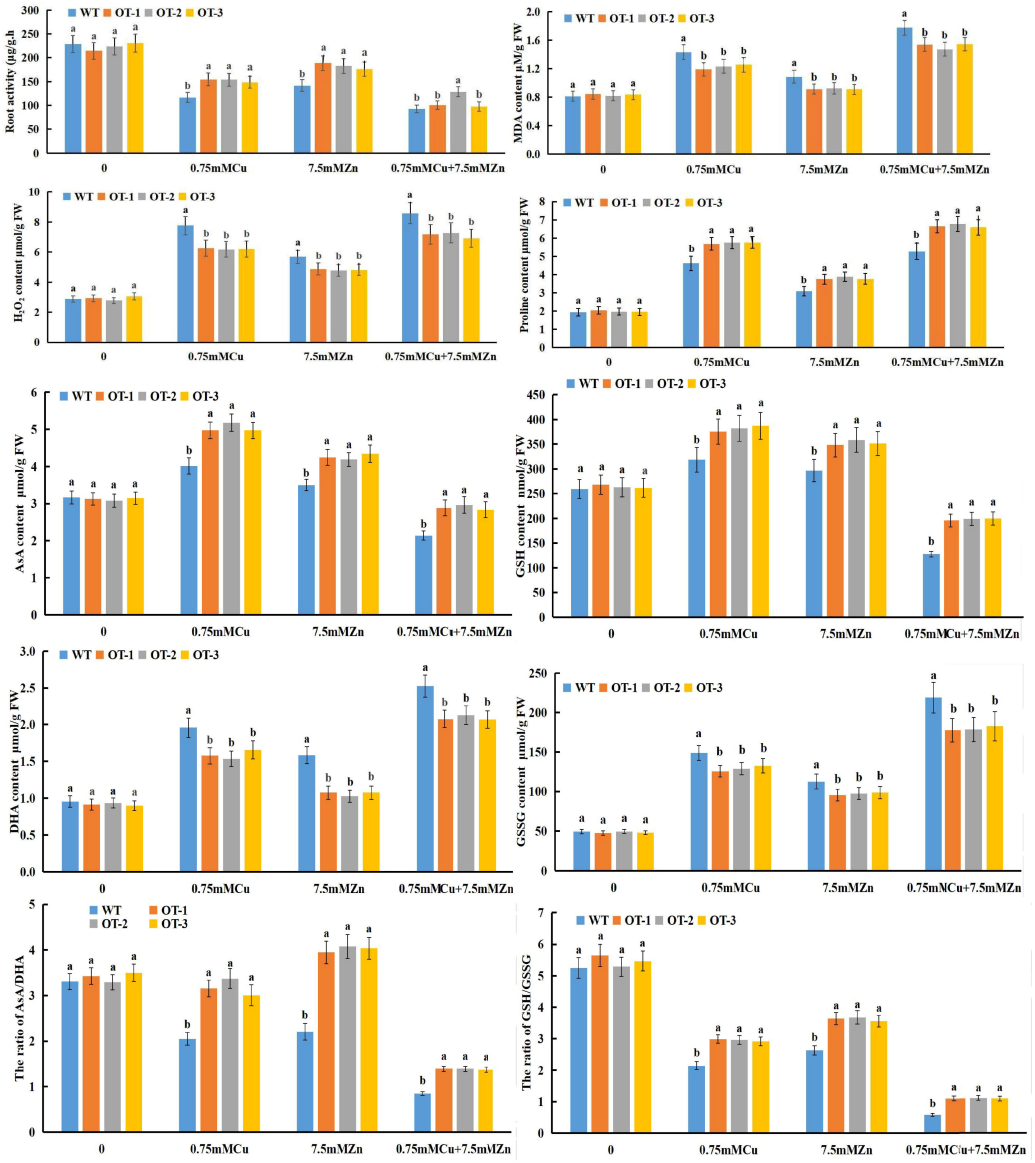
Quantitative analysis of various physiological indexes, including root activity, MDA, H_2_O_2_, Proline, AsA, GSH, DHA, GSSG content and AsA/DHA, GSH/GSSG ratio in WT and OT rice under normal and Cu, Zn stress conditions. Different letters indicate significant differences (p<0.05) between the OT and WT plants under the same conditions.

The roots were affected the most under heavy metal stress (**Figure 4**), and the OT and WT seedlings experienced different degrees of damage when exposed to Cu, Zn stress (**Figure 5**). Compared with that of the control group, the root activity of the OT-treated group decreased by 14.5%/30.8% when it was subjected to Zn/Cu stress, and there were significant differences between the two genotypes. However, the root activity reduced to the maximum level when these seedlings were subjected to Cu+Zn stress, and only the activity of the OT-2 line was significantly different from that of the WT line (**Figure 5**). Malondialdehyde (MDA) and H_2_O_2_ were induced by Cu, Zn stress (**Figure 5**), and the MDA content was remarkably reduced by 21.2%, 18.9% and 12.13%, respectively, compared with that of the WT with Zn, Cu and Cu+Zn treatment (**Figure 5**), whereas the H_2_O_2_ content increased by 69%, 134% and 173% respectively, under Zn, Cu and Cu+Zn stress compared with that the CK (**Figure 5**). In addition, the proline content was significantly higher in the OT (increased by 24.14%, 23.05% and 26.58%) than that in the WT under the stressed conditions (Cu, Zn and Cu+Zn stress, respectively) (**Figure 5**).

The response of the ascorbate-glutathione (AsA-GSH) cycle to Cu, Zn stress in the OT is characterized in **Figure 5**. Compared with that under the control conditions, the AsA content of the OT/WT increased by 34.41%/17.72% and 19.96%/10.05% during Cu, Zn stress, respectively, whereas it decreased by 18.45%/34.42% when it was subjected to Cu+Zn stress. Compared with the WT, all of the OTs presented higher AsA level under the three stress conditions, and the greatest increase AsA content was detected in the Cu+Zn treatment, reached 21.78% (**Figure 5**). As represented in **Figure 5**, GSH and AsA contents had similar results, decreasing during Cu+Zn exposure, and increasing with Cu or Zn individual treatment. As shown in **Figure 5**, the Dehydroascorbate (DHA), Glutathione disulfide (GSSG) contents of the OT and WT seedlings increased under Cu, Zn and Cu+Zn stressful conditions, and the greatest increase was found under the Cu+Zn treatment. However, compared with the WT seedlings, the OT seedlings presented significantly lower DHA and GSSG contents under all three stress conditions. The changes in the AsA, GSH, DHA and GSSG contents of the OT and WT seedlings were conductive to reducing the AsA/DHA and GSH/GSSG ratios (**Figure 5**) after exposure to Cu, Zn single or combined stresses, except for the increased ratio of AsA/DHA in the OT seedlings under Zn stress compared with those under normal conditions.

### 3.9 Assays of antioxidant enzyme activities and the expression of antioxidant-related genes of OT involved in Cu, Zn stress

**Figure 6:**
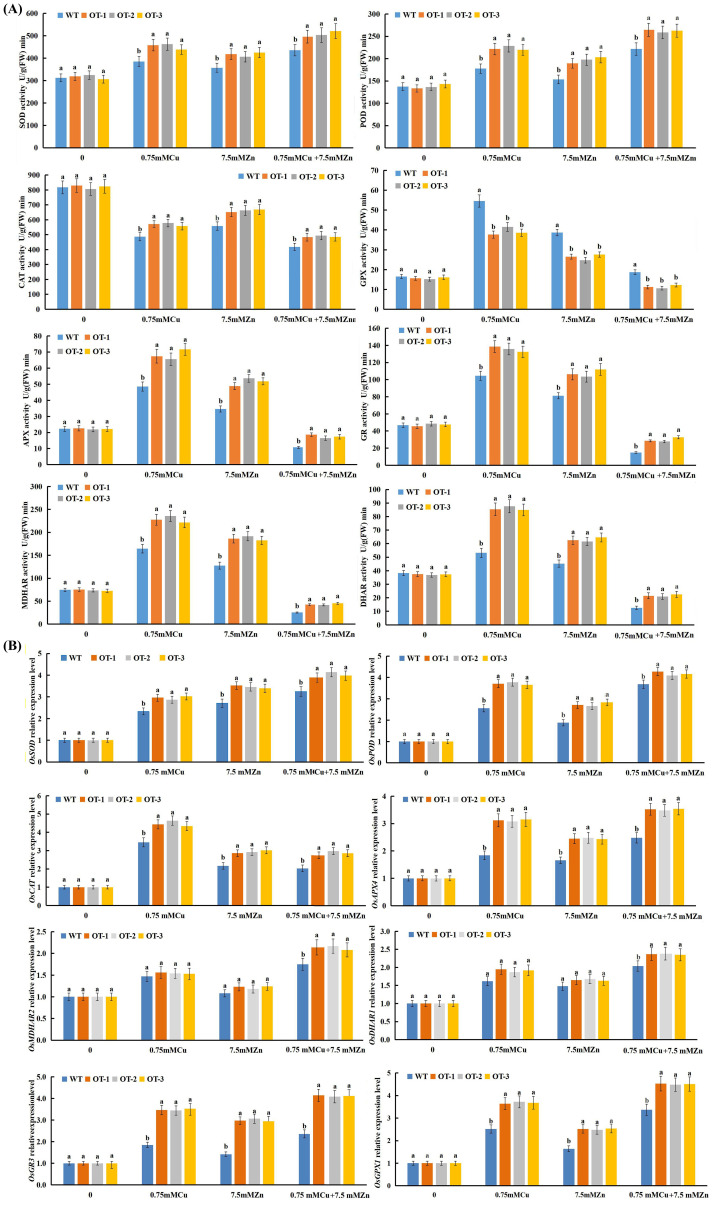
Effect of Cu, Zn on the activities of antioxidative enzymes **(A)** and on the expression level of antioxidative enzyme-encoding genes **(B)**. Data presented are mean ± SE (n=3), different letters indicate significant differences (p<0.05) between the OT and WT plants under the same conditions.

The activities of antioxidative enzymes such as superoxide dismutase (SOD, EC1.15.1.1), peroxidase (POD, EC1.11.1.7), catalase (CAT, EC1.11.1.6), glutathioneperoxidase (GPX), ascorbate peroxidase (APX), glutathione reductase (GR), monodehydroascorbate reductase (MDHAR) and dehydroascorbate reductase (DHAR) were measured in the OTs and WTs exposed to Cu, Zn and Cu+Zn stress conditions (**Figure 6A**). In both the WT and OT rice seedlings, the activities of SOD and POD in response to the Cu, Zn and Cu+Zn treatments improved compared with those in the control, and significantly greater activities were detected in the OT than in the WT (**Figure 6A**) among the three stress treatments. Notably, there was a clear reduction in the CAT activity of the OT and WT plants under all three stress conditions (**Figure 6A**). This might be because the removal of H_2_O_2_ is carried out by APX and GPX, so an increase in CAT activity is not required. Compared with those of the control, the same increasing tendencies were observed for GPX, APX, GR, MDHAR and DHAR activities in both the OT and WT seedlings exposed to Cu, Zn stress, whereas a decreasing tendency was detected under Cu+Zn stress condition (**Figure 6A**). In conclusion, all of the results revealed greater activities of antioxidant enzymes in the OT lines than in the WT. These findings suggested that OT strengthened the ability of the plants to scavenge ROS and maintain ROS homeostasis by increasing the activities of antioxidant enzymes.

To evaluate the regulatory role of *VvOPR1* in gene expression, through which it could improve tolerance to Cu, Zn stress, several antioxidative enzyme-encoding genes were selected for qRT-PCR analysis (**Figure 6B**). The expression of all of the genes showed no significant difference between the WT and OT seedlings under controlled conditions. Conversely, the expression levels of all antioxidative enzyme-encoding genes were significantly greater in the OT and WT seedlings than in the control seedlings when they were exposed to Cu, Zn, Cu+Zn stress. The expression levels of *OsSOD*, *OsPOD*, *OsCAT*, *OsAPX4*, *OsGR3* and *OsGPX1* in the OTs were significantly higher than those in the WT seedlings under either Cu, Zn or Cu+Zn stress conditions (**Figure 6B**). *OsSOD* and *OsPOD* showed the highest expression levels under Cu+Zn stress condition (**Figure 6B**), while the highest expression level of *OsCAT* was observed in the Cu-treated seedlings (**Figure 6B**). The expression pattern of antioxidative enzyme-encoding genes involved in the AsA-GSH cycle (*OsAPX4*, *OsMDHAR2*, *OsDHAR1*, *OsGR3* and *OsGPX1*) was similar to that of *OsPOD* when exposed to Cu, Zn, Cu+Zn stresses (**Figure 6B**), and the highest expression of these genes was also induced by Cu+Zn stress. Compared with those in the control seedlings, the expression levels of *OsMDHAR2* and *OsGR3* were higher in the OT and WT seedlings under Cu, Zn stress conditions, but no significant differences were detected between the OT and WT seedlings (**Figure 6B**).

### 3.10 *VvOPR1* stimulated the ABA-*d*ependent stress responsive pathway

**Figure 7:**
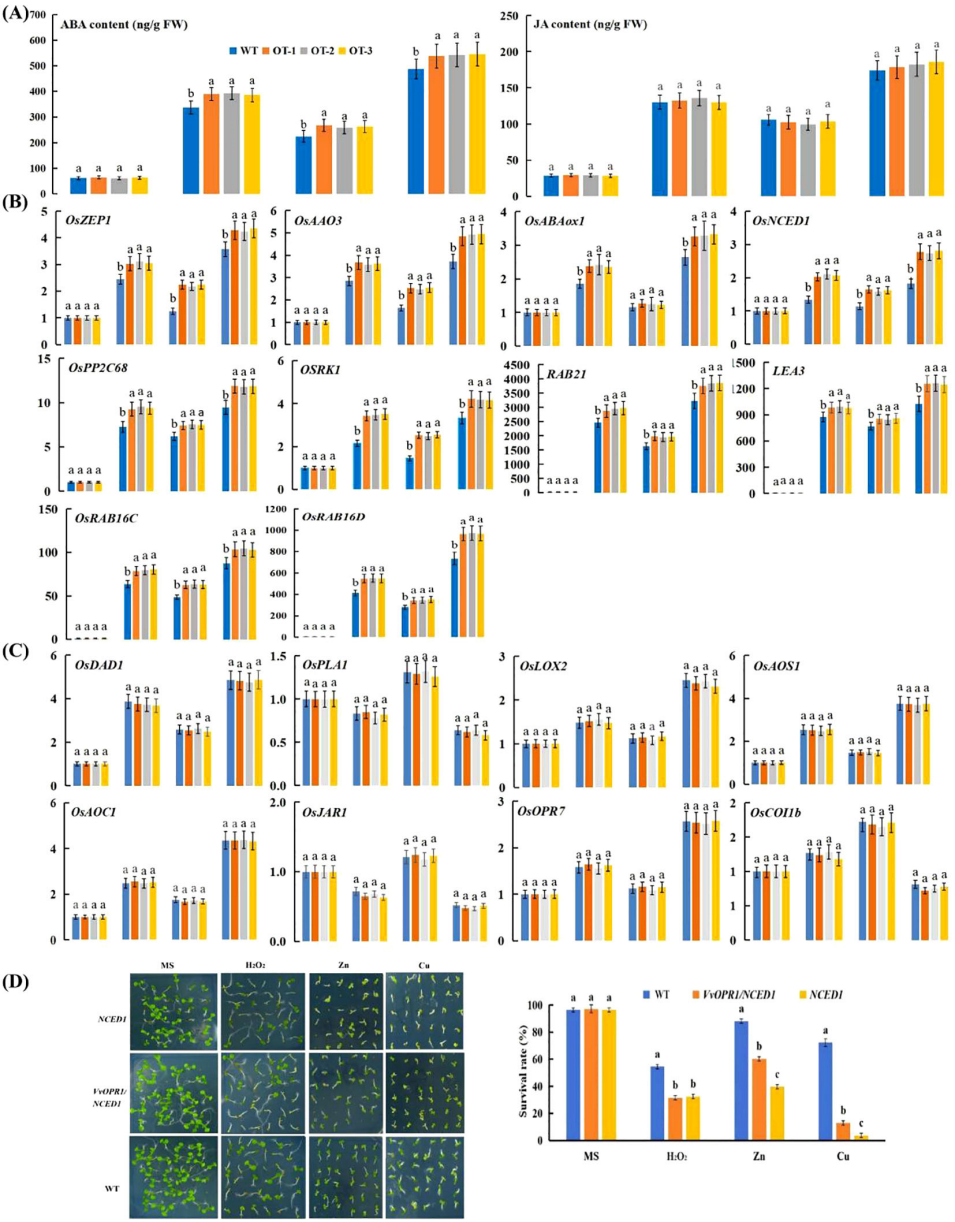
ABA and JA accumulation and the relative expression of ABA and JA- related genes under Cu, Zn stress conditions for five days. **(A)** ABA and JA content in normal and Cu, Zn-treated seedlings. **(B)** The qRT-PCR analysis of the expression of ABA-related genes. **(C)** The qRT-PCR analysis of the expression of JA-related gens. **(D)** The performance of WT, *VvOPR1/NCED1* and *NCED1* mutant *Arabidopsis* seedlings exposure to 1 mM H_2_O_2_, 500 μM Zn and 75 μM Cu stress for two weeks, respectively and the survival rate of seedlings. Each index represents an average of three replicates, and values are means ± standard deviation (SD); and different letters indicate significant differences (p<0.05) in the same index. WT: the wild-type *Arabidopsis* seedlings*, NCED1*: *Arabidopsis NCED1* mutant, *VvOPR1/NCED1*: *Arabidopsis NCED1* mutant lines heterologously expressing *VvOPR1*.

To detect whether the overexpression of *VvOPR1* would induce ABA or JA in OT under Cu, Zn stress conditions, the endogenous ABA and JA levels in OT and WT seedlings were determined. Under control conditions, no significant difference in ABA or JA content was detected between the OT and WT seedlings, whereas the levels of ABA and JA were elevated in both plant types after exposure to Cu, Zn, Cu+Zn stress, and the highest elevation was detected in the Cu+Zn treatment (**Figure 7A**). Specifically, significant differences in ABA levels were detected between the OT and WT under all stressed conditions, whereas no significant differences in JA levels were detected under the same stress conditions.

To further elucidate the molecular mechanism underlying the resistance of OT to Cu, Zn stress, the expression levels of ABA biosynthetic genes (*OsNCED1*, *OsZEP1* and *OsAAO3*), catabolic genes (*OsABA8ox1*), signaling genes (*OsPP2C68* and *OSRK1*) and responsive genes (*RAB21*, *LEA3*, *RAB16C* and *RAB16D*) and JA-related (*OsDAD1*, *OsPLA1*, *OsLOX2*, *OsAOS1*, *OsAOC*, *OsOPR7*, *OsJAR1* and *OsCOI1b*) genes were assayed. The expression levels of ABA-related genes (except for the expression of *OsABA8ox1* under Zn stress conditions) were significantly higher in the OT lines than in the WT seedlings under Cu, Zn stress conditions (**Figure 7B**). As shown in **Figure 7C**, the expression level of *OsCOI1b* in the OT and WT elevated after they were exposed to Cu, Zn stress, but reduced when they were subjected to Cu+Zn stress. The *OsPLA1* and *OsJAR1* genes were downregulated under Cu, Cu+Zn stress conditions but upregulated with Zn treatment. Other JA-related genes, such as *OsDAD1*, *OsLOX2*, *OsAOS1*, *OsAOC* and *OsOPR7* exhibited similar expression patterns: Cu, Zn and Cu+Zn stress all elevated the expression of these genes, with the smallest increase occurring in Zn-treated seedlings, then the expression of these genes increased in the Cu-treated seedlings and peaked under Cu+Zn stress conditions.

To further detect the role of ABA in *VvOPR1*-enhanced Cu, Zn stress, *VvOPR1* was transformed into the *Arabidopsis NCED1* mutant, which is defective in ABA synthesis to construct *VvOPR1/NCED1* lines (**Figure 7D**). Similar results were detected in the WT, *VvOPR1/NCED1* and *NCED1* lines under nonstressed conditions. The survival rate of the *NCED1* mutant was seriously restricted by Cu, Zn stress, and the survival rate was lower by more than 60% compared with that of WT seedlings subjected to Cu stress (**Figure 7D**). *VvOPR1* overexpression significantly increased the survival rate of seedlings subjected to Cu, Zn stress, and the survival rate of *VvOPR1/NCED1* line was 12.96% to 3.7%, 60.19% to 39.81% to those of *NCED1* line under Cu, Zn stress, respectively (**Figure 7D**). However, no significant difference was detected between the *NCED1* mutant and *VvOPR1/NCED1* line under H_2_O_2_ stress.

### 3.11 The accumulation and distribution of Cu^2+^ and Zn^2+^ in OT seedlings

After exposure to 0.75 mM Cu and 7.5 mM Zn for 5 days, the contents of heavy metals drastically increased, and the concentrations of Cu^2+^/Zn^2+^ were significantly lower in the OT than in the WT (**Tables 7**, **8**). Compared with those in the control seedlings, the Cu^2+^ content in the 0.75 mM Cu-treated WT and OT seedlings was nearly 2444- and 2364-fold greater, respectively, than that in the control; whereas the Cu^2+^ content was 2190- and 206-fold greater, respectively under Cu+Zn stress condition. A great increase in Zn^2+^ content was also detected in the Zn-treated seedlings, compared with the control, 849- and 788-fold increases in Zn^2+^ content were detected in the WT and OT seedlings under 7.5 mM Zn stress condition; 724- and 679-fold increases in Zn^2+^ content were detected in the WT and OT seedlings under Cu+Zn stress condition.

**Table 7:** Uptake and concentration of Cu^2+^ in the aerial parts and roots of OT and WT rice seedlings after five days treatment.

Treatment	Cu^2+^ concentration (μg/g DW)				Translocation factor (TF)		Tolerance index (TI) (%)		Accumulation rate (μg/gDW day)	
	The aerial parts		Roots							
	WT	OT	WT	OT	WT	OT	WT	OT	WT	OT
CK	3.47 ± 0.38^aC^	3.58 ± 0.42^aC^	13.75 ± 1.27^aC^	13.65 ± 1.18^aC^	0.25aA	0.26aA	_	_	0.16aC	0.15aC
0.75mM Cu	3672.15 ± 298.14^aA^	3146.64 ± 243.86^bA^	38423.57 ± 3427.63^aA^	37594.13 ± 338.76^aA^	0.10aB	0.08bB	43.01bA	57.64aA	112.09bA	190.28aA
Cu+Zn	3019.53 ± 257.42^aB^	2663.72 ± 214.37^bB^	34707.32 ± 3168.54^aB^	32885.46 ± 3075.48^bB^	0.09aB	0.08bB	32.69bB	36.98aB	46.85bB	87.40aB

Each data point is the mean value ± SD of three replicates, each from three plants. The statistical significance was determined by Duncan’s multiple comparison tests. Different lowercase letters indicate significant differences (P<0.05) between WT and OT rice seedlings in the same row, and different capital letters indicate significant difference (P<0.05)among different treatments in the same column. Total metal accumulation rate expressed as micrograms per gram DW per day.

**Table 8:** Uptake and concentration of Zn^2+^ in the aerial parts and roots of OT and WT rice seedlings after five days treatment.

Treatment	Zn^2+^ concentration (μg/g DW)				Translocation factor (TF)		Tolerance index (TI) (%)		Accumulation rate (μg/gDW day)	
	The aerial parts		Roots							
	WT	OT	WT	OT	WT	OT	WT	OT	WT	OT
CK	23.57 ± 2.16^aC^	23.32 ± 2.43^aC^	42.64 ± 3.86^aC^	42.81 ± 3.75^aC^	0.55aA	0.54aA	_	_	0.64aC	0.65aC
7.5mM Zn	14737.48 ± 1269.35^aA^	11408.65 ± 1076.45^bA^	41526.58 ± 3675.58^aA^	40745.72 ± 3574.37^aA^	0.35aB	0.28bB	62.67bA	79.75aA	350.97aA	343.71aA
Cu+Zn	10496.35 ± 985.31^aB^	8401.43 ± 765.28^bB^	37485.72 ± 3215.63^aB^	36527.88 ± 3185.38^bB^	0.28aC	0.23bC	33.22bB	39.61aB	75.58bB	140.15aB

Each data point is the mean value ± SD of three replicates, each from three plants. The statistical significance was determined by Duncan’s multiple comparison tests. Different lowercase letters indicate significant differences (P<0.05) between WT and OT rice seedlings in the same row, and different capital letters indicate significant difference (P<0.05)among different treatments in the same column. Total metal accumulation rate expressed as micrograms per gram DW per day.

The distribution of Cu^2+^/Zn^2+^ taken up was not homogeneous, and a much higher proportion remained in the roots than was transported into the aerial parts. The proportion of Cu^2+^ in the roots versus the aerial parts was 79.85% versus 20.15%, 91.28% versus 8.72% and 92% versus 8% in WT under the control, Cu and Cu+Zn stress conditions, respectively; 79.22% versus 20.78%, 92.28% versus 7.72% and 92.51% versus 7.49% in the OT under the same conditions. In contrast to the Cu^2+^ distribution, a higher Zn^2+^ proportion was detected in the aerial parts, ranging from 18.7% (the proportion of Zn^2+^ in the OT treated with Cu+Zn stress) to 35.6% (the proportion of Zn^2+^ in the WT under control conditions).

In terms of the translocation factor (TF), under Cu, Zn stress conditions, both the OT and WT lines presented the reduced TFs compared with those under the control conditions. As a consequence of *VvOPR1* overexpression, the distribution of Cu^2+^, Zn^2+^ in rice seedlings was altered, reducing the proportion of Cu^2+^, Zn^2+^ accumulation in the aerial parts of the OT and WT seedlings under Cu, Zn stress. TF values of Cu^2+^, Zn^2+^ in the OT were lower than those in the WT, but the TF value of Cu^2+^ was far below the TF value of Zn^2+^ (**Tables 7**, **8**). The tolerance index (TI) of OT, which is based on root length, was higher than that of WT under all three treatment conditions, indicating that the sensitivity of WT to Cu, Zn stress was greater than that of OT. When exposed to 0.75 mM Cu and 0.75 mM Cu+7.5 mM Zn, the accumulation rates (ARs) of Cu^2+^ in the OT lines were 1.7- and 1.87-fold higher than those in the WT, respectively. While AR in OT was 0.98 and 1.85 fold for Zn^2+^ compared to WT when these seedlings were treated with 7.5 mM Zn or 0.75 mM Cu+7.5 mM Zn, respectively.

## 4 Discussion

*OPR*s are multigene families that can be identified from various plants, and comparative genomics approaches have been used to analyze *OPR* gene families in different plant species (Liu et al., 2020; Stintzi and Browse, 2000; Li et al., 2011). However, no comprehensive studies have focused on the grapevine *OPR* family, and this study was the first to systematically investigate the *VvOPR* family.

### 4.1 *VvOPR1* is an *OPRI* gene mapped to grapevine chromosome 18

In this study, nine *VvOPR*s were identified in grapevine. The rooted maximum-likelihood phylogenetic tree revealed two *OPR* subfamilies in grapevine following the classification system (Montanini et al., 2007), and all of the *VvOPR*s except *VvOPR3* were clustered with sub.I (**Figure 1B**). *VvOPR* genes usually contain 4~6 exons and 3~5 introns according to the *VvOPR* exon/intron structure analyses (**Figure 1C**), and these results were similar to the gene structures of *ZmOPR* and *TaOPR* (Zhang et al., 2005; Mou et al., 2019). A combination of the phylogenetic (**Figure 1B**) and genetic structure analysis (**Figure 1C**) revealed that most of the *VvOPR* genes within a subfamily showed a similar exon/intron structures. *VvOPR4* in sub.I contained the most introns (**Figure 1C**), indicating that the intron loss events occurred during the structural evolution of the *OPR* gene family (Li et al., 2009). As shown by the diversity of VvOPR protein-conserved motifs, the number and the position of *VvOPR* motifs in each subfamily are clearly conserved (**Figure 1C**), with VvOPR1 in sub.I lacking motif 9 and 10; analogously, the motif loss events were also detected in sub.II, and VvOPR3 lacks motif 7, and the absence of this motif is crucial for the secondary structure of proteins (Xin et al., 2017). In general, paralogous genes may have new biological functions relative to their ancestor genes (Guo et al., 2008), so we concluded that *VvOPR1* might function in conjunction with the associated homologous genes *AtOPR1*,*2*.

The number of *OPR* members in the *VvOPR* family differed from that in other representative plants (**Table 1**), possibly because grapevine has undergone whole-genome duplications during its evolutionary history. Gene duplication is one of the primary driving forces in the evolution of genomes (Moore and Purugganan, 2004; Cannon et al., 2004), hence, it is highly possible that the gene duplication led to *VvOPR* gene family expansion, and that new genes contributed to the new structures and new biological functions. Therefore, we deduce that *VvOPR1* might develop some new functions that are different from *AtOPR1,2.*

### 4.2 *VvOPR1*, a novel gene related to abiotic stress tolerance in grapevine, plays a positive role in Cu, Zn stress tolerance

The expression of *OPR*s in dicots and monocots was found to be tissue specific (Liu et al., 2020; Stintzi and Browse, 2000; Li et al., 2011; Biesgen and Weiler, 1999; Mou et al., 2019; Xin et al., 2017; Creelman and Mullet, 1995). Here, *VvOPR1* showed the highest expression level in roots, followed by stems and leaves, and the expression profiles of *VvOPR1* under abiotic stress treatments (Cu, Zn, H_2_O_2_ and ABA stresses) (**Figure 5**) were much different from those of *GhOPR* (Liu et al., 2020), *ZmOPR* (Zhang et al., 2005) and *TaOPR* (Wang et al., 2016a; Dong et al., 2013). Therefore, based on the specific stress inductive patterns of *VvOPR1* suggest that *VvOPR1* is involved in the response to Cu, Zn stress.

Research on *OPR*s participating in development and response to various abiotic and biotic stresses in some plants has been conducted thoroughly (Bosch et al., 2014; Dave et al., 2011). In this study, the growth retardation was observed in the OT and WT seedlings under Cu, Zn stress, however, the OT seedlings showed obvious phenotypic changes compared with the WT seedlings, manifested as higher generation rates, longer shoot and root lengths and higher fresh weights (**Figure 4**). The growth reduction raised with increasing levels of Cu, Zn, which induced toxicity at elevated concentrations, and the impact on root elongation was greater than that on shoot growth (**Figure 4**); similar results have been reported for durum wheat (Michaud et al., 2007; Ghavri and Singh, 2012; Bibi et al., 2006; Shu et al., 2002). In *Phaseolus vulgaris*, Cu toxicity disrupted the capacity of cells to remove oxidatively damaged proteins by inhibiting the ubiquitin proteasome pathway in embryonic stages thus inhibiting seed germination (Karmous et al., 2014). Seed germination which is very sensitive to the external medium is regulated by changes in the cellular redox status (Nanda and Agrawal, 2016), and the addition of metal further aggravates the microenvironment, causing damage to proteins and leading to a reduction in germination (El-Maarouf-Bouteau and Bailly, 2008). In this study, the relatively high germination rate of OT under Cu, Zn stress conditions might have been due to decreased accumulation of oxidatively damaged proteins. The Cu, Zn-induced growth reduction has also been observed in *Spirodela polyrhiza* (Upadhyaya and Panda, 2010), *Withania somnifera* (Khatun et al., 2008) and *Sorghum bicolor* (Soudek et al., 2014).

### 4.3 Possible ABA-mediated mechanisms for Cu, Zn stress tolerance in OT

#### 4.3.1 A positive role of *VvOPR1* in Cu, Zn stress-responsive ABA signaling

ABA is a vital component of the abiotic stress response, and increasing ABA synthesis and/or limiting ABA catabolism frequently occurs in plant in response to abiotic stress (Jakab et al., 2005). In response to abiotic stresses, the ABA content dramatically increases in transgenic plants overexpressing some ABA biosynthesis-related genes to cope with the stress (Yue et al., 2012; Hwang et al., 2010). Similar expression levels of ABA biosynthetic (*OsNCED1*, *OsZEP1* and *OsAAO3*) and catabolic (*OsABA8ox1*) genes were detected in the OT and WT seedlings under normal conditions, which indicated that *VvOPR1* alone may not be sufficient to regulate ABA biosynthesis. However, the higher expression levels of these genes in the OT seedlings exposed to Cu, Zn stress clearly indicated that the possible role of *VvOPR1* in Cu, Zn stress-induced ABA-biosynthesis, along with additional factors. In this study, although the expression levels of both ABA biosynthetic (*OsNCED1*, *OsZEP1* and *OsAAO3*) and catabolic (*OsABA8ox1*) genes were elevated, the speed of ABA synthesis may be much faster than that of catabolism, so the ABA content was elevated in Cu-, Zn-treated seedlings, suggesting its involvement in the induction of protective mechanisms against excess Cu, Zn toxicity. This finding is in agreement with a previous study in which ABA was found to increase under Cu (Vishwakarma et al., 2017), Zn stress (Wang et al., 2014). The ABA content was significantly higher in the OT lines than in the WT, indicating the possible role of *VvOPR1* induced ABA accumulation in response to Cu, Zn stress and an acceleration in ABA-dependent pathway caused by *VvOPR1* overexpression is initiated from a burst in ABA synthesis.

ABA triggers a signaling cascade that regulates a suite of abiotic stress responsive genes (Nakashima et al., 2009). Here, the expression levels of the early ABA signaling genes (*OsPP2C68* and *OSRK1*) and late ABA-responsive genes (*RAB21*, *OsLEA3*, *RAB16C* and *RAB16D*) in Cu-, Zn-treated seedlings were obviously elevated compared with those in the control condition, and the higher expression level in OT (**Figure 7B**) provides firm evidence for a positive role of *VvOPR1* in stress-responsive ABA signaling. Overall the results suggested that the overexpression of *VvOPR1* induced ABA synthesis and enhanced ABA content in OT seedlings under Cu, Zn stress conditions, thus, leading to increased expression levels of ABA signaling and responsive genes. However, further study is necessary in order to better understand how component genes involved in ABA signal transduction mediate Cu, Zn stress through the induction of gene expression induction.

#### 4.3.2 VvOPR1 confers Cu, Zn tolerance by enhancing antioxidation capacity

Excess Cu, Zn often cause the generation of ROS, and excess ROS in the cell are associated with extensive lipid peroxidation (Deng et al., 2010; Olmos et al., 2003; Cho et al., 2012), which generates a range of toxic breakdown products, such as α, β unsaturated aldehydes (Trotter et al., 2006; Esterbauer, 1993). Here, MDA accumulated in WT and OT seedlings during Cu, Zn stress, albeit to a lesser extent in the OT lines than in the WT, significantly (**Figure 5**). High lipid peroxidation in rice seedlings might be the result of ROS-induced oxidative stress or increased lipoxygenase activity caused by Cu, Zn stress. Similar results have also been reported in *Triticum aestivum* (Panda et al., 2003) in response to Zn and in *Brassica juncea* (Radic et al., 2010) in response to Cu. These results indicate that higher protection against oxidative damage in OT might be due to less ROS generation, less peroxidation, and the ability of *VvOPR1* to alleviate ROS damage.

In addition to counteracting the toxicity of ROS-induced lipid peroxidation, the direct neutralization of ROS has been proposed as a component of stress tolerance (Mittler, 2002). The OYZ family is believed to protect the cell against the damaging effects of lipid peroxidation products, and the function of OYE in yeast also appears to be to reduce the level of ROS present (Fitzpatrick et al., 2003). In this study, compared with those in WT seedlings, the reduced H_2_O_2_ in Cu, Zn-treated OT may be attributed to the increased levels of antioxidative enzymes involved in scavenging this oxidant (Thounaojam et al., 2012). Previous studies have shown that CAT has a high capacity but low affinity, whereas POD has a high affinity for H_2_O_2_ (Mittler et al., 2002). The decrease in CAT activity indicated that CAT was not required for the elimination of H_2_O_2_ in Cu-, Zn-stressed rice plants and that POD is the major effective H_2_O_2_-scavenging enzyme for reducing H_2_O_2_ in OT cells under Cu, Zn stress. In addition, APX has a greater affinity for H_2_O_2_ than dose CAT and it may play a more critical role in the regulation of ROS (Nanda and Agrawal, 2016), and increased APX activity (**Figure 6A**) under Cu stress has been previously reported (Tewari et al., 2006). Higher AsA (**Figure 5**) and increased SOD activity (**Figure 6A**) are associated with the detoxification of superoxide radicals to H_2_O_2_ which may be responsible for the increased activity of APX under Cu, Zn stress conditions (Li et al., 2013; Lukatkin et al., 2014). This result is in accordance with the findings of Drazkiewicz et al. (2003), where the ASH-GSH cycle plays an important role in reducing the toxic effect of Cu. Thus *VvOPR1* overexpression increased the content of several low-molecular weight nonenzymatic compounds (**Figure 5**), increased the activities of several ROS scavenging enzymes (**Figure 6A**) and the expression of their encoding genes (**Figure 6B**) and there is some evidence that *VvOPR1* promotes the efficiency of ROS scavenging to affect the removal of ROS and alleviate their deleterious effects.

ABA can increase the transcription and activity of ROS network genes, and defects in this network can also disrupt the expression of ABA and stress responsive genes (Miao et al., 2006). Moreover, the *VvOPR1/NCED1 Arabidopsis* mutant did not rescue the sensitivity of the *NCED1* mutant to H_2_O_2_ (**Figure 7D**). Hence, it could be concluded that the enhanced efficiency of ROS scavenging promoted by *VvOPR1* expression may be, at least in part, mediated by an acceleration of ABA synthesis and an upregulation of relevant signaling pathways.

#### 4.3.3 *VvOPR1* enhanced Cu, Zn tolerance by reducing Cu^2+^, Zn^2+^ accumulation and translocation

In this work the behavior of WT and OT seedlings with respect to Cu, Zn tolerance, accumulation and translocation were compared. Both the OT and WT showed a prominent Cu, Zn accumulation in the roots under Cu, Zn stress conditions, and their accumulation differed significantly between the OT and WT seedlings. Compared with the OT seedlings, the WT showed a greater ability than OT to accumulate and translocate the metal to the aerial parts of the seedlings. In addition, the TI and AR in the OT in terms of Cu^2+^, Zn^2+^ were detected (**Tables 7**, **8**). On the basis of the dry biomass of the total plants, the TI and AR revealed that the tolerance to Cu, Zn was much greater than that of the WT. Together, these results confirmed that *VvOPR1* has a considerable potential to alleviate Cu^2+^, Zn^2+^ induced damage by reducing Cu^2+^, Zn^2+^ accumulation and translocation. The reduction translocation of Cu^2+^, Zn^2+^ from roots to the aerial organs could reduce the damaging effects of theses pollutants on leaf physiology and biochemistry.

A previous study has reported that ABA can reduce heavy metal stress by affecting heavy metal transport to the aerial parts (Perfus-Barbeoch et al., 2002). It is possible that ABA-induced stomatal closure suppressed of transpirational flow, resulting in a restriction of root-to-aerial translocation of metals (Bücker-Neto et al., 2017). Exogenous ABA application reduced the transport of Cd, Ni from the roots to the leaves, resulting in greater metal accumulation in the roots (Rubio et al., 1994), and in response to treatment with CdCl_2_, the ABA content rapidly increased in the leaves and roots of Cd-tolerant cultivar rice seedlings (Hsu and Kao, 2003). These findings are consistent with that the lower TF (**Tables 7**, **8**) of OT seedlings, which accumulated higher ABA (**Figure 7A**) content, and were more tolerant of Cu, Zn stress than the WT. To date, studies are still in progress to characterize the OT seedlings with respect to the biochemical and molecular processes involved in the accumulation and translocation of Cu^2+^, Zn^2+^ to the aerial organs.

### 4.4 *VvOPR1* did not disturb JA synthesis or signaling machinery

Recently, JA has been shown to be effective in improving plant tolerance to heavy metal stress (Yan et al., 2015; Piotrowska et al., 2009; Ahmad et al., 2017). In this study, the increase in JA levels in the leaves of the OT and WT seedlings in response to the Cu, Zn treatments was consistent with the increase in JA levels after Cu and Cd treatment (Maksymiec et al., 2005). The increase in JA caused by Cu, Zn stress could involve up-regulation of some JA biosynthesis genes, such as *OsDAD1*, *OsPLA1*, *OsLOX2*, *OsAOS1*, *OsAOC1* and *OsOPR7* (**Figure 7C**). These results suggested that JA is involved in Cu, Zn stress, however, no significant difference was defected in JA content or the expression level of JA synthesis genes was detected between OT and WT seedlings, so we concluded that neither the expression of JA synthesis genes nor the endogenous JA level was dependent on *VvOPR1* overexpression (**Figure 7C**). In addition, similar results were detected for the expression levels of the JA signaling related genes *OsJAR1* and *OsCoI1b* (**Figure 7C**). These findings indicate that *VvOPR1* dose not regulate JA synthesis or signaling pathways.

In combination, we like to conclude that *VvOPR1* may be an effective gene for improving Cu, Zn stress tolerance. Here, we first synthesized and characterized the characterization the *OPR*I gene *VvOPR1*, which regulates the expression of ABA biosynthesis and catabolism genes thus leading to increased endogenous ABA accumulation under Cu, Zn stress conditions, subsequently promoting the ABA signaling pathway, and reducing Cu, Zn accumulation and translocation. Moreover, *VvOPR1* overexpression reduced the production of ROS (such as reduced H_2_O_2_ and MDA) and increased the ROS-scavenging system to confer tolerance to Cu, Zn stress. Therefore, we concluded that *VvOPR1* may be an effective gene for improving Cu, Zn stress tolerance in plants. In addition, the function of *VvOPR1* may lie in the metabolism of trans-(+)-OPDA, with consequent effects on the activities of the (ABA-dependent responsive and/or ROS) signaling pathway which this molecule mediates. Moreover, the presence of *VvOPR1* did not induce any changing in JA synthesis or signaling pathways.

## 5 Conclusions

In summary, nine *VvOPR* genes were identified from grapevine genome and classified into two subfamilies. and the evolution was relatively conservative in the group; chromosome mapping confirmed that *VvOPR* family genes were only distributed on chromosome 11 and 18, gene structure analysis identified that the structure of *VvOPR*s was highly conservative, especially the conservative motif 6, 4, 2 and 3 were shared by all genes; promoter analysis revealed that the promoter region of OPR genes were rich in *cis*-element which response to growth and development, hormone signals and adversity. In addition, the results revealed that Cu, Zn had a relatively low toxicity, but could do damage to plants at some concentrations. Cu, Zn stress decreased the biomass, and photosynthetic activities due to increasing ROS. While *VvOPR1* overexpression alleviated Cu, Zn stress and reduced the growth restriction. These biochemical mechanistic findings suggested in OT seedlings, *VvOPR1* enhanced the photosynthetic capacity, promoted ABA synthesis and the ABA-dependent stress response pathway, improved the activities of ROS scavengers and the expression levels of their encoding genes, increased the accumulation of proline, AsA, GSH, while alleviated MDA and H_2_O_2_ accumulation. Moreover, *VvOPR1* reduced Cu^2+^, Zn^2+^ accumulation, translocation. Together, ABA may play a crucial role in the response *VvOPR1*-overexpressing seedlings to Cu, Zn stress, *VvOPR1* responds to Cu, Zn stress in an ABA-dependent manner to enhance tolerance to Cu, Zn stress, and this effect appears to be quite independent from JA synthesis or JA signaling. Despite the progress achieved, further work is needed to determine how ABA guides adaptation under Cu, Zn stress conditions.

## Data Availability

The datasets presented in this study can be found in online repositories. The names of the repository/repositories and accession number(s) can be found in the article/**Supplementary Material**.
