# Supplementary material for: Genome-wide analysis of OPR family genes in Vitis vinifera and the role of VvOPR1 in copper, zinc tolerance
Source: Front Plant Sci. 2025 Feb 26;16:1509472. doi: 10.3389/fpls.2025.1509472 (PMC11897507; doi:10.3389/fpls.2025.1509472)
Supplement: Supplementary file 1 [file Table1.docx]

**TABLE S1 The specific primers used for qRT-PCR analysis**

| **Gene name** | **Forward (5'-3')** | **Reverse (5'-3')** |
| --- | --- | --- |
| *VvOPR1* | 5'-GATCCTGTTCTGGGTTACACT-3' | 5'-CTGAACAAGTACGGTCTGCTG-3' |
| *VvActin* | 5'-CCCCATGCTATCCTTGC-3' | 5'-AGGCAGCTCATAGTTCTTCTC-3' |
| *OsUBQ5* | 5'-ACCACTTCGACCGCCACTACT-3' | 5'-ACGCCTAAGCCTGCTGGTT-3' |
| *OsSOD* | 5'-CGCTCAGAGCCTCCTCTTT-3' | 5'-CTCCTGGGGTGGAGACAAT-3' |
| *OsPOD* | 5'-AGGCCCAGTGCTHCAMCTTC-3' | 5'-TTGGTGTAGTAGGCGTTGTC-3' |
| *OsCAT* | 5'-GCACAGTTTGACAGGGAGCG-3' | 5'-GAACACCAGGAGCACGGAGA-3' |
| *OsAPX4* | 5'-GGAAGGGCACATCCTGAAAG-3' | 5'-TCTTGTGCGATTCAGCGTAGTC-3' |
| *OsMDHAR2* | 5'-GCCATTCTTCTACTCCAGGGTCT-3' | 5'-CCTTGGGCTGCTGTTTGTGA-3' |
| *OsDHAR1* | 5'-AGGTGCCCTACGAGATGAAGC-3' | 5'-AATCCATTTGCCATCACCACC-3' |
| *OsGR3* | 5'-GGCTGTGGGTGATGTAACGAA-3' | 5'-GGATGGAGAAAACAGCACAAGG-3' |
| *OsGPX1* | 5'-TTGCATTGAGCACTTGGAAC-3' | 5'-AGGGGCAAAGTGATGCAGTA-3' |
| *OsZEP1* | 5'-ATAGATGATGGCAACAAGGTAA-3' | 5'-TCAATGTCAGGAGGCACAA-3' |
| *OsNCED1* | 5'-CACTCCCTTCTCATTCCC-3' | 5'-AGCCCTTGTTCAGGTTAA-3' |
| *OsAAO3* | 5'-CGCCTGGTAAAGTGTCTA-3' | 5'-AATTGCTCCTTGAGTGGT-3' |
| *OsABA8ox1* | 5'-CCAAGAACCCCAACGTGTTC-3' | 5'-CGGGCTGGACACCATCA-3' |
| *OsPP2C* | 5'-CGCAGCTCCGACAACATCT-3' | 5'-GCTGGGTGACACTCTCTCTACAAG-3' |
| *OSRK1* | 5'-AGTACACCAAGCAGGTGAAGCA-3' | 5'-GCAACAGCAAAGCTTGAACTCA-3' |
| *RAB21* | 5'-CACACCACAGCAAGAGCTAAGTG-3' | 5'-TGGTGCTCCATCCTGCTTAAG-3' |
| *LEA3* | 5'-GCCGTGAATGATTTCCCTTTG-3' | 5'-CACACCCGTCAGAAATCCTCC-3' |
| *OsRAB16C* | 5'-TTCCCGGCCAGCACTAAAT-3' | 5'-AAACTGCACGTACATCACGACAT-3' |
| *OsRAB16D* | 5'-CGGGTAAACAATAAAGTCGTGATG-3' | 5'-GCGCACTTACATACAGTGCTACGT-3' |
| *OsPLA1* | 5'-GCCAAAGCAGTCATCTCGTC-3' | 5'-CCAGTCACTCGTAGGGCTAACA-3' |
| *OsDAD1* | 5'-ACCTCATCGACGGGTTCAC-3' | 5'-CGGTTCACGTACTCCTTCTTCA-3' |
| *OsLOX2* | 5'-GCATCCCCAACAGCACATC-3' | 5'-AATAAAGATTTGGGAGTGACATATTGG -3' |
| *OsAOS1* | 5'-CAAGGACTTCGTCGTGCTC-3' | 5'-TCCGTATCCGTACAAGCTGA-3' |
| *OsAOC* | 5'-GAGGCTTCTTGGTAGTAGGTGGA-3' | 5'-CGTAGTGGCGGTCGTTGTAGT-3' |
| *OsOPR7* | 5'-GGATGTAAATGTACTGCGGGAT-3' | 5'-ACAATCTGTGCTGATGACCCA-3' |
| *OsJAR1* | 5'-ACCTCCAGTTGGCTGTTGAG -3' | 5'-CCTTGCTGTTTGATGGGTTC-3' |
| *OsCOI1b* | 5'-GTAATGTTGGGGAAACAGATG-3' | 5'-AAGCTTGCTCACTGAAGCAACAA-3' |

**TABLE S2. Dry weight in** **the aerial parts and roots of OT and WT rice seedlings after 5 days Cu^2+^ treatment**

| Treatment | Dry weight (mg/plant) | | | |
| --- | --- | --- | --- | --- |
|  | The aerial parts | | Roots | |
|  | WT | OT | WT | OT |
| CK | 212.78±14.15^Aa^ | 211.54±13.92^Aa^ | 117.46±3.56^Aa^ | 116.96±3.78^Aa^ |
| 0.75mMCu | 127.86±3.24^Bb^ | 175.24±4.47^Ab^ | 63.86±1.14^Bb^ | 83.24±3.16^Ab^ |
| Cu+Zn | 81.92±3.18^Bc^ | 104.76±3.46^Ac^ | 45.74±2.73^Bc^ | 68.3±1.47^Ac^ |

Note: Each data point is the mean value ± SD of three replicates, each from 3 plants. The statistical significance was determined by Duncan’s multiple comparison tests. Different capital letters indicate significant differences (P＜0.05) between WT and OT rice seedlings in the same row, and different lowercase letters indicate significant difference (P＜0.05）among different treatments in the same column.
